# Dual activity of PNGM-1 pinpoints the evolutionary origin of subclass B3 metallo-*β*-lactamases: a molecular and evolutionary study

**DOI:** 10.1080/22221751.2019.1692638

**Published:** 2019-11-21

**Authors:** Jung Hun Lee, Masayuki Takahashi, Jeong Ho Jeon, Lin-Woo Kang, Mineaki Seki, Kwang Seung Park, Myoung-Ki Hong, Yoon Sik Park, Tae Yeong Kim, Asad Mustafa Karim, Jung-Hyun Lee, Masayuki Nashimoto, Sang Hee Lee

**Affiliations:** a National Leading Research Laboratory of Drug Resistance Proteomics, Department of Biological Sciences, Myongji University, Yongin, Republic of Korea; b Research Institute for Healthy Living, Niigata University of Pharmacy and Applied Life Sciences, Niigata, Japan; c Department of Biological Sciences, Konkuk University, Seoul, Republic of Korea; d Marine Biotechnology Research Center, Korea Institute of Ocean Science & Technology, Busan, Republic of Korea

**Keywords:** Antimicrobial resistance, subclass B3 metallo-*β*-lactamase, tRNase Z, dual activity, structure and evolutionary origin

## Abstract

Resistance to *β*-lactams is one of the most serious problems associated with Gram-negative infections. *β*-Lactamases are able to hydrolyze *β*-lactams such as cephalosporins and/or carbapenems. Evolutionary origin of metallo-*β*-lactamases (MBLs), conferring critical antibiotic resistance threats, remains unknown. We discovered PNGM-1, the novel subclass B3 MBL, in deep-sea sediments that predate the antibiotic era. Here, our phylogenetic analysis suggests that PNGM-1 yields insights into the evolutionary origin of subclass B3 MBLs. We reveal the structural similarities between tRNase Zs and PNGM-1, and demonstrate that PNGM-1 has both MBL and tRNase Z activities, suggesting that PNGM-1 is thought to have evolved from a tRNase Z. We also show kinetic and structural comparisons between PNGM-1 and other proteins including subclass B3 MBLs and tRNase Zs. These comparisons revealed that the B3 MBL activity of PNGM-1 is a promiscuous activity and subclass B3 MBLs are thought to have evolved through PNGM-1 activity.

## Introduction

Antibiotic resistance is a steadily increasing global problem which could lead to a fundamental upheaval in clinical care with the potential to return us to the pre-antibiotic era [1–3]. The production of *β*-lactamases, a group of enzymes that confer *β*-lactam resistance in Gram-negative bacteria, is now one of the major barriers in treating Gram-negative infections [4–6]. *β*-Lactamases are classified according to their catalytic mechanisms into serine *β*-lactamases (classes A, C, D) and metallo-*β*-lactamases (MBLs, class B enzymes further divided into three subclasses B1–B3) [7,8]. There are functional and structural similarities between serine *β*-lactamases and penicillin-binding proteins (PBPs), and so serine *β*-lactamases are thought to have evolved from a PBP [8,9]. Due to the functional and structural differences between MBLs and serine *β*-lactamases, the MBLs, hydrolyzing almost all *β*-lactam antibiotics and thereby representing a critical antibiotic resistant threat, are thought to have evolved from a protein other than a PBP [9–12]. However, to date this ancestor remains unknown. Interestingly, we discovered PNGM-1 (Papua New Guinea Metallo-*β*-lactamase), the novel subclass B3 metallo-*β*-lactamase, in deep-sea sediments that predate the antibiotic era [13].

Subclass B1 and B3 MBLs require two zinc ions for maximum *β*-lactamase activity, whereas the subclass B2 MBLs require only one [14]. MBLs belong to an ancient MBL superfamily [15,16]. Members of this superfamily include MBLs, tRNase Zs from all three domains and so forth [15,17–20]. Amino acid sequence identity between the members is low (less than 5%), but structural features, i.e. *αββα*-fold or MBL fold, and the unique metal binding motif (HXHXDH) are shared [18,19].

A member of the MBL superfamily, tRNase Z, is a tRNA processing enzyme which removes the 3′ trailer from pre-tRNA [21]. Most tRNase Zs cleave pre-tRNA immediately downstream of a discriminator nucleotide (nt), onto which the CCA residues are added to produce mature tRNA. tRNase Z can cleave unstructured single-strand RNAs that are unrelated to pre-tRNA *in vitro* [22]. The tRNase Z catalytic centre is formed by the major five well conserved residues [His-48, His-50, Asp-52, His-53, and His-222 in the case of *T. maritima* tRNase Z (prokaryotic tRNase Z^S^)] together with two zinc ions [23]. Residues Asp-52 and His-222 are thought to directly contribute as donors during the catalytic proton transfer [23].

To date, there is a study [15] or hypothesis [24] on the evolution of MBLs. The structure-based phylogeny [15] showed that MBLs and rubridoxin oxidoreductase (an MBL fold protein without *β*-lactamase activity, a member of MBL superfamily) descended from a common ancestor. However, this ancestor and the origin of MBLs remains unknown. Due to the structural similarity between the two halves (*αβ* and *βα* domains) of MBLs, there was a proposal that MBLs might be arisen by a gene-duplication event [24]. However, there is no evidence showing the event. PNGM-1 (a subclass B3 MBL) identified in deep-sea sediments that predate the antibiotic era [13] showed unique characteristics as follows. First, PNGM-1 contains 386 amino acids (AAs), making it longer by about 40–100 AAs in length than the length (280 to 342 AAs) of amino acid sequences of subclass B3 MBLs. Second, while checking on the NCBI database using the BLASTP programme (https://blast.ncbi.nlm.nih.gov/Blast.cgi?PAGE=Proteins), we found that PNGM-1 showed high sequence identities (74 and 71%, respectively) to the MBL fold metallo-hydrolases (members of MBL superfamily; GenBank accession numbers: WP_146397269 and TDI33721, respectively) of *Planctomycetes* bacterium CA13 and *Acidobacteria* bacterium identified from the marine sediment metagenome. The annotated sequences of, and limited information about, the two MBL fold proteins were released at the GenBank database from March to August 2019. Therefore, knowledge of the enzymatic functions of these MBL fold metallo-hydrolases remains incomplete. However, PNGM-1 showed a low sequence identity (≤18%) to MBLs with *β*-lactamase activity. These results suggest that PNGM-1 is able to possess an enzymatic activity (other than *β*-lactamase) which is one of the various biological functions of the MBL superfamily, including DNA or RNA processing [16].

In this study, we found that in addition to *β*-lactamase activity, PNGM-1 possesses endoribonuclease (tRNase Z) activity on both pre-tRNA substrates and on small unstructured single-strand RNA substrates. Our functional, phylogenetic, and structural analyses of PNGM-1 and their comparison with the properties of other MBLs (proteins containing the *αββα*-fold with *β*-lactamase activity) and structurally representative MBL fold proteins (proteins having *αββα*-fold without *β*-lactamase activity) of the MBL superfamily, reveal the evolution of tRNase Z to subclass B3 MBLs through the dual activity of PNGM-1.

## Materials and Methods

### Strains and plasmids

*Escherichia coli* BL21 (DE3) and plasmid-containing *E. coli* strains were used for all cloning and expression studies. The pET-28a(+)/His_6_-PNGM-1 plasmid has been described previously [13]. The strains and plasmids used in this study are listed in Table S1.

### Construction of PNGM-1 mutants by site-directed mutagenesis

Site-directed mutagenesis was carried out using a QuikChange II Site-Directed Mutagenesis Kit (Stratagene, Agilent Technologies, Santa Clara, CA, USA) according to the manufacturer’s instructions. H91A, H93A, D95A, H96A, and H257A mutants were generated, with the template (pET-28a(+)/His_6_-PNGM-1) and primer sequences listed in Table S2. After verifying the DNA sequences, the plasmids, [pET-28a(+)/His_6_-PNGM-1(H91A), pET-28a(+)/His_6_-PNGM-1(H93A), pET-28a(+)/His_6_-PNGM-1(D95A), pET-28a(+)/His_6_-PNGM-1(H96A), and pET-28a(+)/His_6_-PNGM-1(H257A)], were individually transformed into *E. coli* BL21 (DE3) cells.

### Cloning of bla_AIM-1_, bla_GOB-18_, bla_FEZ-1_, and three tRNase Z genes

Six DNA templates encoding *bla*_AIM-1_ gene (GenBank ID: AM998375), *bla*_GOB-18_ gene (GenBank ID: DQ004496), *bla*_FEZ-1_ gene (GenBank ID: Y17896), and three tRNase Z genes from *Bacillus subtilis* (Bs-tRNase Z, GenBank ID: WP_101502431), *E. coli* (Ec-tRNase Z, GenBank ID: Q47012), and *T. maritima* (Tm-tRNase Z, GenBank ID: NP_228673), which are codon optimized for *E. coli*, were synthesized and purchased from IDT (Integrated DNA Technologies, Coralville, IA, USA). The DNA templates were amplified by PCR using suitable primer pairs (Table S2). The amplified DNA and pET-30a(+) vector (Novagen, Madison, Wisconsin, USA) were double-digested with *Nde*I and *Xho*I, with digested DNA then ligated into the digested vector. After verifying the DNA sequences, the plasmids, pET-30a(+)/His_6_-*bla*_AIM-1_, pET-30a(+)/His_6_-*bla*_GOB-18_, pET-30a(+)/His_6_-*bla*_FEZ-1_, pET-30a(+)/His_6_-Bs-tRNase Z, pET-30a(+)/His_6_-Ec-tRNase Z plasmid, and pET-30a(+)/His_6_-Tm-tRNaseZ, were individually transformed into *E. coli* BL21 (DE3) cells.

### Preparation of PNGM-1, PNGM-1 mutants, AIM-1, GOB-18, FEZ-1, and tRNase Zs

Each of the histidine-tagged proteins, PNGM-1, five PNGM-1 mutants (H91A, H93A, D95A, H96A, and H257A), AIM-1, GOB-18, FEZ-1, Bs-tRNase Z, Ec-tRNase Z, Tm-tRNase Z, and human Δ30 tRNase Z^L^ (lacking the N-terminal 30 amino acids) were prepared as previously described [13,21,25].

### Steady-state kinetic analysis

Kinetic assays were conducted at 30°C with a Shimadzu UV-1650PC spectrophotometer (Shimadzu Corp.). *β*-Lactam hydrolysis was detected by monitoring the change in absorbance using the characteristic molar extinction coefficient of each substrate: cefoxitin (Δ*ε*_270 nm_ = −8,380 M^−1^ cm^−1^); ceftazidime (Δ*ε*_265 nm_ = −10,300 M^−1^ cm^−1^); cefotaxime (Δ*ε*_264 nm_ = −7,250 M^−1^ cm^−1^); imipenem (Δ*ε*_278 nm_ = −5,660 M^−1^ cm^−1^); meropenem (Δ*ε*_298 nm_ = −9,530 M^−1^ cm^−1^); and ertapenem (Δ*ε*_295 nm_ = −10,940 M^−1^ cm^−1^). The assays were conducted in 50 mM MES [2-(N-morpholino)ethanesulfonic acid] (pH 7.0) buffer which contained the enzyme, 100 μM ZnCl_2_ and 100 μg ml^–1^ bovine serum albumin. Steady-state kinetic constants were determined by fitting the initial rates (in triplicate) directly to the Michaelis–Menten equation using nonlinear regression with the programme DynaFit [26]. To determine whether PNGM-1 was halophilic or halotolerant to NaCl (from 0 to 2 M), enzyme activity was measured using benzylpenicillin and cephalothin as the substrate, as previously described [27].

### Construction of the phylogenetic tree

Multiple sequence alignments of PNGM-1 with 82 representative enzymes of subclasses B1, B2, and B3 MBLs from the Beta-Lactamase DataBase (http://bldb.eu/), structurally representative MBL fold proteins [a Zn-dependent hydrolase of *Thermotoga maritima* (Tm), another Zn-dependent hydrolase of *T. maritima* (Tm-1), lactonase from *T. maritima* (Tm-Lac), N-acyl homoserine lactone hydrolase from *Bacillus thuringiensis* (AiiA), 4-pyridoxolactonase from *Mesorhizobium japonicum* MAFF 303099 (PDLA), alkylsulfatase from *Pseudomonas aeruginosa* PAO1 (SdsA1), sulfur dioxygenase of *Arabidopsis thaliana* (ATSD), a teichoic acid phosphorylcholine esterase from *Streptococcus pneumoniae* (CbpE), a methyl parathion hydrolase from *Pseudomonas* sp. strain WBC-3 (Pah), a human glyoxalase II (Gox), *β*-CASP metallo-*β*-lactamase family nuclease from *Methanothermobacter thermautotrophicus* (MTH1203), and tRNase Zs] [18], and two MBL fold metallo-hydrolases [Pl (WP_146397269) from *Planctomycetes* bacterium CA13 and Ac (TDI33721) of *Acidobacteria* bacterium] were performed with MAFFT (Multiple Alignment using Fast Fourier Transform) in the Geneious software (v11.1.5, Biomatters, http://www.geneious.com). The phylogenetic tree was constructed by the neighbor-joining method using the Molecular Evolutionary Genetics Analysis X software (MEGA, version X) [28]. Accession numbers of all enzymes used for phylogenetic analysis are listed in Table S3.

### Structure determination of PNGM-1

Crystallization and X-ray diffraction data collection of PNGM-1 was carried out as previously published [29]. All X-ray diffraction data were integrated and scaled using the *DENZO* and *SCALEPACK* crystallographic data-reduction routines [30].

The PNGM-1 structure was solved by the SAD method using SeMet PNGM-1. The interpretable electron density was obtained at 2.3 Å resolution for the SeMet PNGM-1 data set using the single wavelength SAD protocol of AUTO-RICKSHAW, an automated crystal structure determination platform [31] in the *P*2_1_ space group. The native PNGM-1 structure was determined by molecular replacement with *MOLREP* [32] using the SeMet PNGM-1 structure (Table S4).

### Preparation of RNA and DNA substrates

Total RNA of the human multiple myeloma cell line KMM-1 was extracted with RNAiso Plus (Takara Bio, Shiga, Japan). A 24-nt RNA, usRNA1 (5′-GAGUGACUACCUCCAAGGCCCUUU-3′), a 22-nt RNA, usRNA9 (5′-GCCUGGCUGGCUCGGUGUAUUU-3′), and a 24-nt DNA, usDNA1 (5′-GAGTGACTACCTCCAAGGCCCTTT-3′), were chemically synthesized with a 5′-6-carboxyfluorescein dye and subsequently purified by high-performance liquid chromatography. These were obtained from JBioS (Saitama, Japan). An 84-nt human pre-tRNA^Arg^, (5′-GGGCCAGUGGCGCAAUGGAUAACGCGUCUGACUACGGAUCAGAAGAUUCCAGGUUCGACUCCUGGCUGGCUCGGUGUAAGCUUU-3′), was synthesized using T7 RNA polymerase (Takara Bio, Shiga, Japan) from a corresponding synthetic DNA template, and subsequently 5′-labeled with fluorescein as previously described [33].

### In vitro RNA cleavage assay

Various RNA substrates and a negative control DNA substrate were mixed with PNGM-1 in 50 μl of a 0.2–1 mM MES (pH 6.8) buffer or with tRNase Z in 50 μl of 4 mM Tris-HCl (pH 8.0) buffer in the presence of 5–50 mM MgCl_2_ or MnCl_2_ at 25–80°C for 30–90 min. The reaction products for the total RNA of the human cell line KMM-1 were analyzed by microfluidics-based automated electrophoresis with the Agilent 2100 Bioanalyzer (Agilent Technologies, CA, USA) according to the manufacturer’s protocol. The reaction products for the other substrates were separated on a 20% polyacrylamide 8 M urea gel or in the case of the reaction products for the DNA substrate with MnCl_2_, a 20% polyacrylamide native gel. After resolution of the reaction products, the gel was analyzed with a Gel-Doc imager (Bio-Rad Laboratories, CA, USA). Total RNA isolated from the human cell line KMM-1 was incubated with 15 μM PNGM-1 in the presence of 10 mM Mg^2+^ or Mn^2+^ at 37°C for 60 min, and analyzed by microfluidics-based automated electrophoresis. Three substrates [usRNA1 (24 nt), usRNA9 (22 nt), and usDNA1 (24 nt)], which were 5′-labeled with 6-carboxyfluorescein, were incubated with 15 μM PNGM-1 in the presence of 10 mM Mg^2+^ at 50°C for 30–90 min, and the products were analyzed by denaturing polyacrylamide gel electrophoresis.

## Results

### *β*-Lactamase activity of PNGM-1 and PNGM-1 mutants

The subclass B3 MBL, PNGM-1, has recently been discovered from the metagenomic library of deep-sea sediments from the Edison seamount that predates the antibiotic era [13]. While PNGM-1 has low amino acid sequence identity with other B3 MBLs, it does possess the unique metal binding motif (H_116_XH_118_XD_120_H_121_; numbering according to the BBL scheme [8]) that is only present in subclass B3 MBLs. To test the *β*-lactamase (MBL) activity of PNGM-1, four active-site mutants, two at the first zinc-binding site [H91A (H116A, numbering according to the BBL scheme [8]) and H93A (H118A)] and two at the second zinc-binding site [D95A (D120A) and H96A (H121A)], were generated by site-directed mutagenesis (Tables S1 and S2).

To investigate the *β*-lactam-hydrolyzing activity of the four PNGM-1 mutants against *β*-lactam antibiotics, the catalytic properties of purified PNGM-1 and the four PNGM-1 mutants were assessed (Table 1). Kinetic assays revealed that all four mutants (H91A, H94A, D95A, and H96A) were unable to hydrolyze *β*-lactam antibiotics including extended-spectrum cephalosporins (ceftazidime and cefotaxime) and carbapenems (meropenem, imipenem, and ertapenem) (Table 1). Substitution of residues in the metal binding motif had the most significant influence on the catalytic properties of PNGM-1 against cephalosporins and carbapenems. Therefore, these results indicated that PNGM-1 possesses *β*-lactamase (MBL with its ability to hydrolyze carbapenems) activity, and the four residues (H91, H94, D95, and H96) are essential for exerting *β*-lactamase activity.

**Table 1.** Kinetic parameters of PNGM-1 and five PNGM-1 mutants for various *β*-lactams.

Substrate and parameter	PNGM-1^a^	H91A	H93A	D95A	H96A	H257A^b^
Cefoxitin						
* K*_m_ (μM)	4.3 ± 0.1	ND^c^	ND	ND	ND	3.8 ± 0.1
* k*_cat_ (s^–1^)	0.0015 ± 0.0001	ND	ND	ND	ND	0.0007 ± 0.0001
* k*_cat_/*K*_m_ (M^–1^ s^–1^)	(3.5 ± 0.2) × 10^2^	ND	ND	ND	ND	(1.8 ± 0.2) × 10^2^
Ceftazidime						
* K*_m_ (μM)	15.9 ± 0.2	ND	ND	ND	ND	15.4 ± 0.1
* k*_cat_ (s^–1^)	0.0025 ± 0.0001	ND	ND	ND	ND	0.0011 ± 0.0001
* k*_cat_/*K*_m_ (M^–1^ s^–1^)	(1.5 ± 0.2) × 10^2^	ND	ND	ND	ND	(1.0 ± 0.2) × 10^2^
Cefotaxime						
* K*_m_ (μM)	3.3 ± 0.1	ND	ND	ND	ND	3.1 ± 0.1
* k*_cat_ (s^–1^)	0.0027 ± 0.0002	ND	ND	ND	ND	0.001 ± 0.0002
* k*_cat_/*K*_m_ (M^–1^ s^–1^)	(8.1 ± 0.1) × 10^2^	ND	ND	ND	ND	(3.0 ± 0.1) × 10^2^
Meropenem						
* K*_m_ (μM)	1.9 ± 0.1	ND	ND	ND	ND	ND
* k*_cat_ (s^–1^)	0.0008 ± 0.0001	ND	ND	ND	ND	ND
* k*_cat_/*K*_m_ (M^–1^ s^–1^)	(4.2 ± 0.1) × 10^2^	ND	ND	ND	ND	ND
Imipenem						
* K*_m_ (μM)	2.0 ± 0.1	ND	ND	ND	ND	ND
* k*_cat_ (s^–1^)	0.0011 ± 0.0001	ND	ND	ND	ND	ND
* k*_cat_/*K*_m_ (M^–1^ s^–1^)	(5.5 ± 0.2) × 10^2^	ND	ND	ND	ND	ND
Ertapenem						
* K*_m_ (μM)	1.9 ± 0.1	ND	ND	ND	ND	ND
* k*_cat_ (s^–1^)	0.0009 ± 0.0001	ND	ND	ND	ND	ND
* k*_cat_/*K*_m_ (M^–1^ s^–1^)	(4.7 ± 0.2) × 10^2^	ND	ND	ND	ND	ND

^a^Kinetic data for PNGM-1 were identical to data from reference [13].

^b^His-257 of PNGM-1 is located at the position corresponding to His-222 of *T. maritima* tRNase Z.

^c^ND, not detectable.

Data are mean ±s.d. of three assays.

Most of the enzymes from deep sea are halophilic or halotolerant [34]. PNGM-1 exhibited the highest activity in the presence of 50 mM NaCl (Table S5). Moreover, the activity was maintained with up to 2 M NaCl (Table S5). Therefore, PNGM-1 is halotolerant (salt-resistant) to NaCl.

### The insight into the evolutionary origin of subclass B3 MBLs

**Figure 1.**
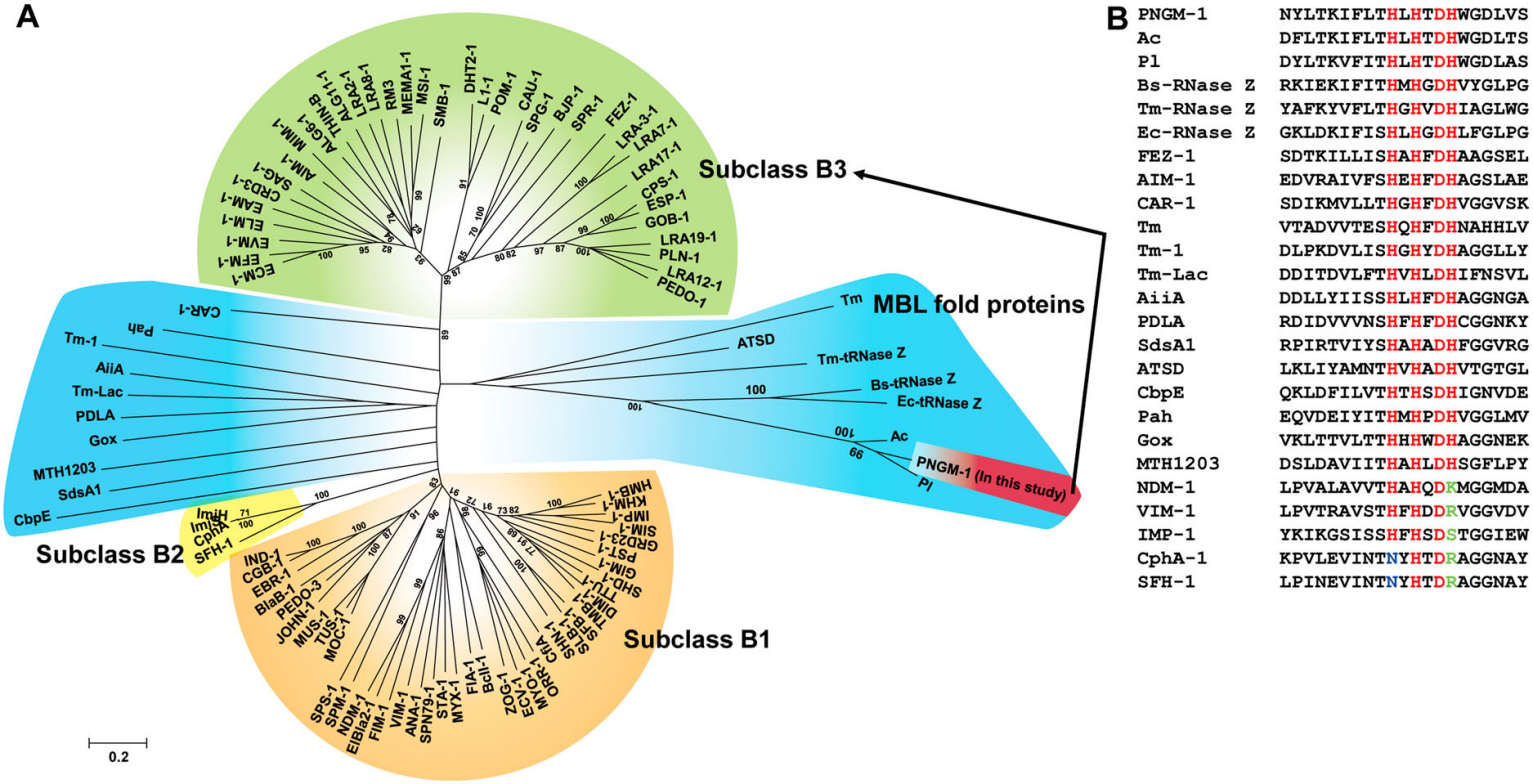
Characteristics of subclass B3 MBL PNGM-1. (A) Neighbor-joining phylogenetic tree of PNGM-1 with all MBL types (B1, B2, and B3 MBL subclasses) and structurally representative enzymes of MBL fold proteins that have diverse functions but no *β*-lactamase activity. Only bootstrap values higher than 50% are shown. Bar, 0.2 substitutions per amino acid site. (B) Multiple sequence alignment of PNGM-1 with representative sequences of B1, B2, and B3 MBLs and MBL fold proteins. The unique metal-binding motif, HXHXDH, is well conserved in subclass B3 MBLs including PNGM-1 and MBL fold proteins, and is indicated with a box. The closed triangle shows the difference between subclass B2 (CphA and SFH-1) and others, with the differing residues highlighted in blue, while the closed square shows the difference between B1 and B2 MBLs (NDM-1, VIM-1, IMP-1, CphA, and SFH-1) and others, with the differing residues highlighted in green. The closed circles highlight residues which are conserved in all MBLs and MBL fold proteins. Accession numbers of all enzymes used for phylogenetic analysis are listed in Table S3.

The evolutionary origin of subclass B3 MBLs has not yet been identified. However, MBLs belong to an ancient MBL superfamily [15,16] that evolved billions years ago [35] and to date, there are more than 80 different types of MBLs in the Beta-Lactamase DataBase (http://bldb.eu/). A phylogenetic analysis was carried out with all MBL types, PNGM-1, 14 structurally representative MBL superfamily members [MBL fold proteins having no *β*-lactamase activity (Table S3) such as Bs-tRNase Z, Tm-tRNase Z, and Ec-tRNase Z [18]], and two MBL fold metallo-hydrolases (Pl and Ac with no functional analysis). Our phylogenetic analysis revealed that (i) PNGM-1 was not grouped with subclass B3 MBLs but grouped with MBL fold proteins (Figure 1(A)); (ii) PNGM-1 was closest to the tRNase Zs of the MBL fold proteins (Figure 1(A)); and (iii) the HXHXDH motif was completely conserved in PNGM-1, the subclass B3 MBLs (AIM-1, GOB-18, and FEZ-1), and MBL fold proteins, but not in subclasses B1/B2 MBLs (Figure 1(B)). These results suggest that PNGM-1 gives us an insight into the evolutionary origin of subclass B3 MBLs.

### Structural similarity between PNGM-1 and tRNase Z

**Figure 2.**
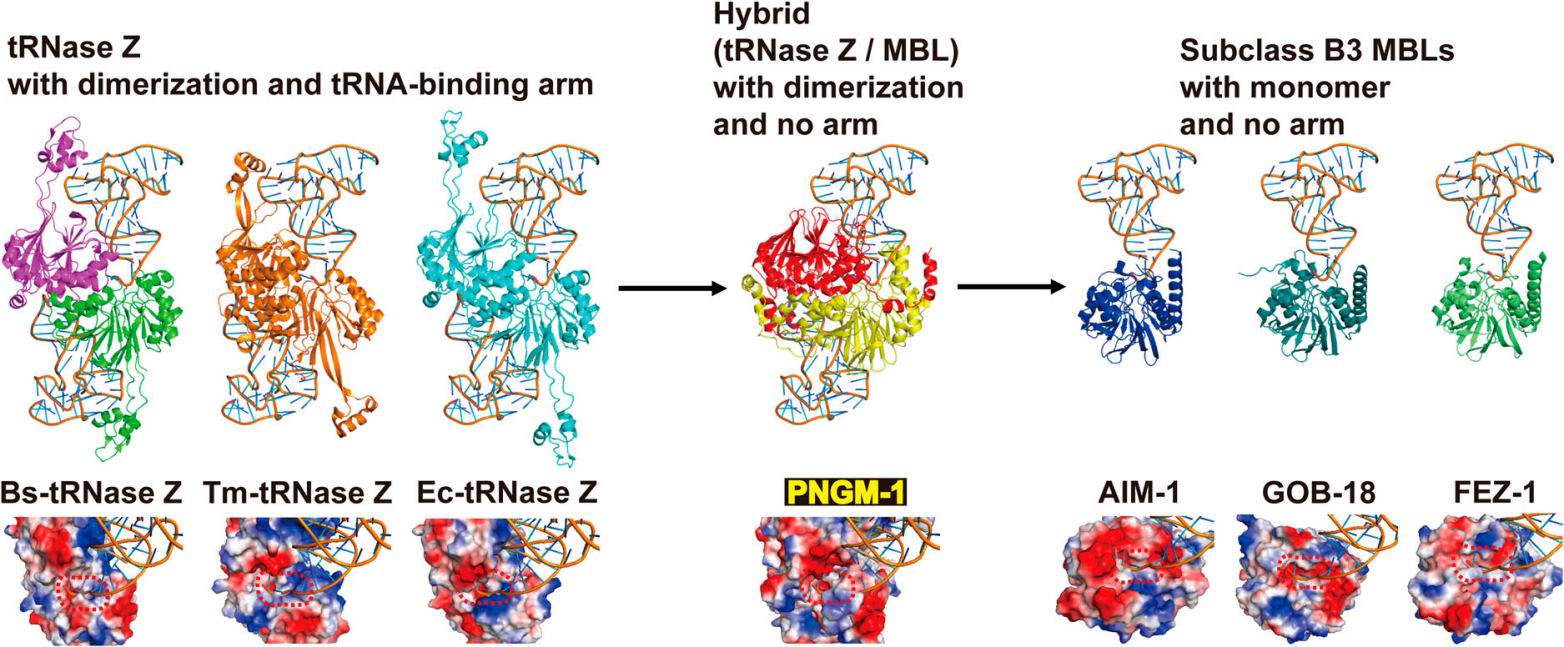
Gradual structural changes of tRNase Zs, PNGM-1 and subclass B3 MBLs. The structure of PNGM-1 [subunit A (red) and B (yellow), PDB entry 6J4N] was compared with those of tRNase Z from *B. subtilis* [Bs-tRNase Z (purple/green), PDB entry 4GCW], *T. maritima* [Tm-tRNase Z (orange/orange), PDB entry 2E7Y], and *E. coli* [Ec-tRNase Z (cyan/cyan), PDB entry 2CBN] and subclass B3 MBLs from *P. aeruginosa* [AIM-1 (blue), PDB entry 4AWZ]*, Elizabethkingia meningoseptica* [GOB-18 (pale green), PDB entry 5K0W], and *Legionella gormanii* [FEZ-1 (lime), PDB entry 5W90]. The tRNA-bound Bs-tRNase Z structure was superimposed on the structures of the enzyme compared and the superimposed tRNA was presented with the enzymes (top row). In all structures, the 3’ end position of superimposed tRNA was bound in the substrate-binding pocket (bottom row). The substrate-binding pocket is marked with a red dashed line.

To examine the evolutionary relationship between PNGM-1 and tRNase Z, we determined the PNGM-1 structure and compared the structure with those of structurally representative tRNase Zs from *B. subtilis*, *E. coli*, and *T. maritima* (Figure 2). All the structures have the characteristic *αββα*-fold of the MBL superfamily with the unique metal binding motif. The core of the PNGM-1 monomer is well superimposed on the tRNase Z monomers of Bs-tRNase Z, Ec-tRNase Z, and Tm-tRNase Z using Coot programme (http://bernhardcl.github.io/coot/), with the root-mean-square-deviation of 2.61 Å in the Cα atom of 178 aligned residues, 2.46 Å in 175 residues, and 2.05 Å in 202 residues, respectively. In addition, comparison of the PNGM-1 structure with all structures in the Protein Data Bank, using DALI server (http://ekhidna2.biocenter.helsinki.fi/dali/), revealed that the highest structural similarity is for Bs-tRNase Z (Dali Z-score: 29.7), followed by Ec-tRNase Z (29.4), Tm-tRNase Z (22.2), and subclass B3 MBLs [AIM-1 (10.0), GOB-18 (9.8), and FEZ-1 (9.6)] (Table S6).

Structures of all tRNase Z enzymes showing dimerization [19] is essential for tRNA binding and tRNase Z activity; tRNA is mostly bound to subunit A with the 3’ end bound to the active site of subunit B (Figure 2). PNGM-1 structure also maintains the dimerization interactions and the dimer interface of PNGM-1 is strictly conserved to that of tRNase Z (Figure S1). Specifically, the dimer interface consists of three helices and a loop: three helices of residues 43–49, 70–82, and 95–106; and a loop of residues 9–18 in Bs-tRNase Z and three helices of residues 71–77, 98–110, and 132–143; and a loop of residues 39–48 in PNGM-1 (Figure S1). When the tRNA-bound Bs-tRNase Z dimer was superimposed on the PNGM-1 dimer, tRNA can be bound to PNGM-1 subunit A without major hindrance and the 3’ end (to be processed) of tRNA is well positioned in the active site of subunit B (Figure 2). The structurally representative subclass B3 MBLs, AIM-1, GOB-18, and FEZ-1, exist as monomers due to the presence of bulge-like structures at the proposed dimer interface which prevents dimerization due to steric hindrance (Figure 2). tRNA is a bulky substrate similar to tRNase Z enzyme monomer. However, the dimerization of subclass B3 MBLs is not necessary for the binding of much smaller *β*-lactam substrates. Therefore, subclass B3 MBLs are unable to interact with tRNA except the 3’ end of tRNA to be processed (Figure 2). tRNase Z has a conserved tRNA-binding arm, which is lost in PNGM-1 and subclass B3 MBLs (Figure 2). This analysis of the superimposed structures of these six enzymes with PNGM-1 showed the gradual change from tRNase Z to subclass B3 MBL (Figure 2). These structural similarities between tRNase Zs and PNGM-1 prompts us to investigate the possibility of PNGM-1 possessing tRNase Z activity.

### PNGM-1 possesses RNase activity

**Figure 3.**
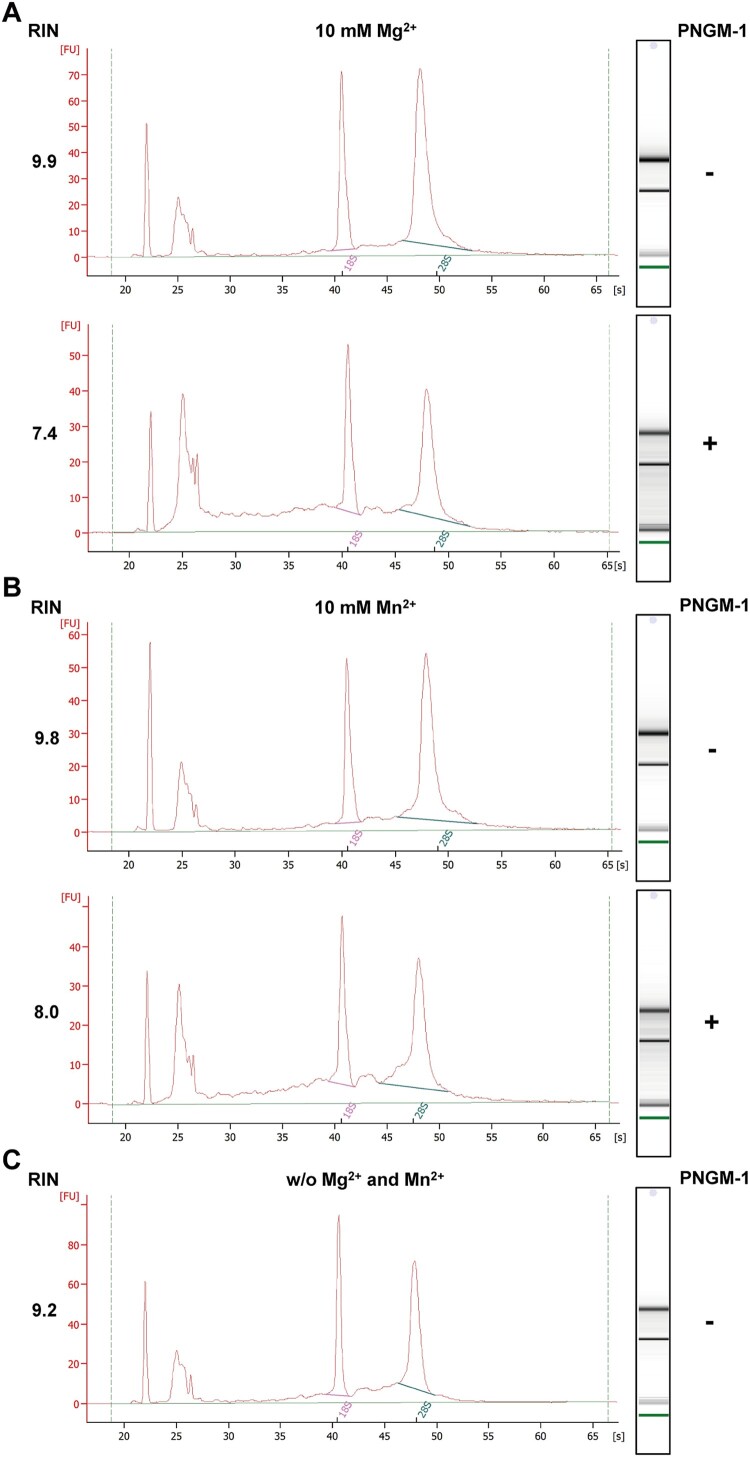
*In vitro* assays for RNase activity of PNGM-1 on total human cell RNA. Total RNA (0.3 μg) of the human cell line KMM-1 was incubated with or without 15 μM PNGM-1 in the presence of 10 mM MgCl_2_ (A) or MnCl_2_ (B)**,** at 37°C for 60 min, and analyzed by microfluidics-based automated electrophoresis. Input RNA without PNGM-1 in the absence of any additional metal ions is shown in (C). An electropherogram and a gel image for each sample are shown. The peaks around 22 and 25 s denote the 25-nt marker (green line on the gel image) and approximately 100-nt RNAs including tRNA, 5S rRNA, and 5.8S rRNA, respectively. 18S, 18S rRNA (pink line); 28S, 28S rRNA (blue line); FU, arbitrary fluorescence units; RIN, RNA integrity number. Experiments in (A)-(C) were repeated at least three times and were reproducible.

To test whether PNGM-1 possesses RNase activity, we examined whether PNGM-1 can degrade total RNA isolated from the human cell line KMM-1. Total RNA was incubated with 15 μM PNGM-1 in the presence of 10 mM Mg^2+^ or Mn^2+^ at 37°C for 60 min, and analyzed by microfluidics-based automated electrophoresis. Both 18S and 28S rRNAs were degraded to smaller RNAs, and RNA species (∼100 nt) accumulated under both conditions (10 mM Mg^2+^ and Mn^2+^) (Figure 3). The RNA integrity numbers in the assays with Mg^2+^ and Mn^2+^ decreased by 25% and 18%, respectively. These results suggest that PNGM-1 possesses a ribonuclease activity.

### PNGM-1 can cleave unstructured RNAs endoribonucleolytically

**Figure 4.**
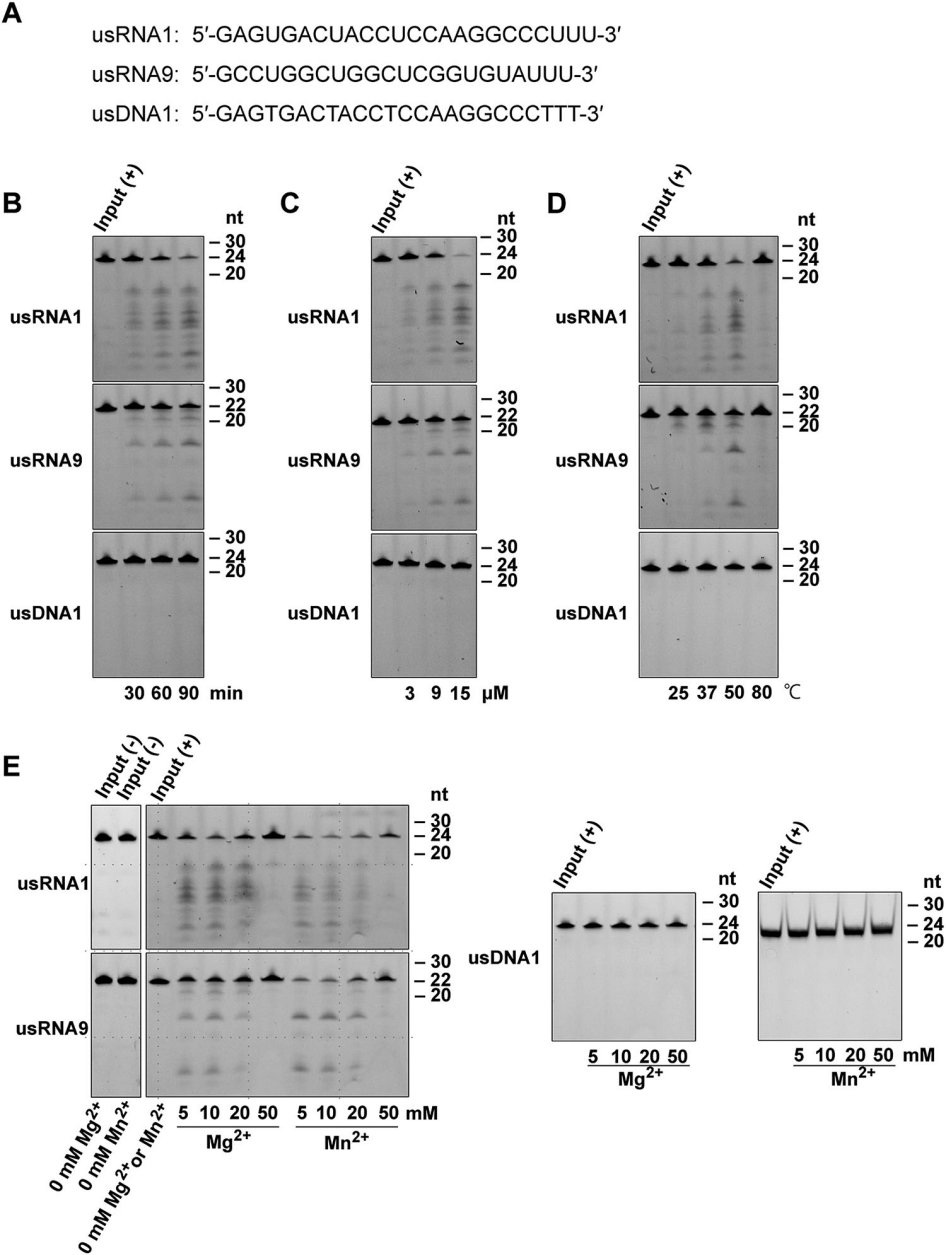
*In vitro* assays for RNase activity of PNGM-1 on small unstructured RNA substrates. (A) The 5′-6-carboxyfluorescein-labeled substrates (usRNA1, usRNA9, and usDNA1) were incubated with PNGM-1, and the products were analyzed on either a 20% polyacrylamide 8 M urea gel or a 20% polyacrylamide native gel. The native gel was used only for analysis of usDNA1 reactions with MnCl_2_. (B) The substrates were incubated with 15 μM PNGM-1 in the presence of 10 mM MgCl_2_ at 50°C for 30, 60, and 90 min. (C) The substrates were incubated with 3, 9, and 15 μM PNGM-1 in the presence of 10 mM MgCl_2_ at 50°C for 90 min. (D) The substrates were incubated with 15 μM PNGM-1 in the presence of 10 mM MgCl_2_ at 25, 37, 50, and 80°C for 90 min. (E) The substrates were incubated without (−) or with (+) 15 μM PNGM-1 in the absence or presence of 5–50 mM MgCl_2_ or MnCl_2_ at 50°C for 90 min. Experiments in (B–E) were repeated at least three times and were reproducible.

Next, we examined PNGM-1 for RNase activity on two small RNA substrates. The unstructured RNAs, usRNA1 (24 nt) and usRNA9 (22 nt), were used as they are cleaved by tRNase Z. An unstructured 24-nt DNA (usDNA1), corresponding to usRNA1 was used as a negative control substrate. These three substrates, which were 5′-labeled with 6-carboxyfluorescein, were incubated with 15 μM PNGM-1 in the presence of 10 mM Mg^2+^ at 50°C for 30–90 min, and the products were analyzed by denaturing polyacrylamide gel electrophoresis. Fragments of various sizes were generated from usRNA1 and usRNA9 but not from usDNA1 by PNGM-1 (Figure 4(A)). The pattern of generated RNA fragments suggests that PNGM-1 possesses an endoribonuclease activity, not an exoribonuclease activity, and has no DNase activity. The amounts of usRNA1 and usRNA9 cleavage products increased in a dose-dependent manner, with the optimal temperature for RNase activity estimated to be around 50°C (Figure 4(B) and (C)). RNase activity was also assessed by varying the concentration (5–50 mM) of MgCl_2_ or MnCl_2_, and the optimal concentrations of Mg^2+^ and Mn^2+^ were estimated to be around 10 mM and 5–10 mM, respectively (Figure 4(D)). It should be noted that PNGM-1 shows no RNase activity without Mg^2+^ or Mn^2+^ ions. This metal ion requirement for the RNase activity would be related to stabilizing the RNA/PNGM-1 interaction, the chemical step of cleavage, or both of these.

### Asp-95 and His-257 are essential for the RNase activity of PNGM-1

**Figure 5.**
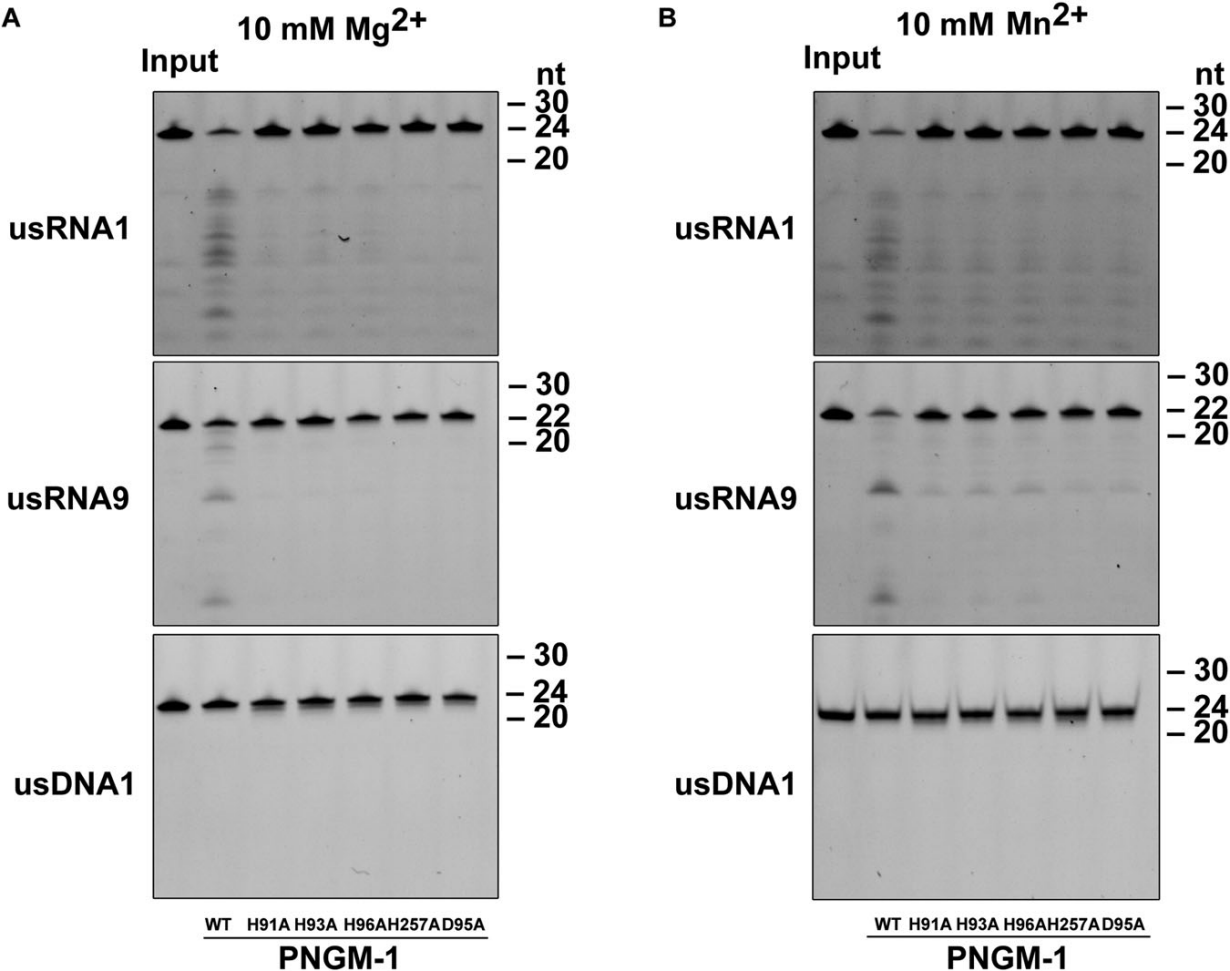
*In vitro* RNA cleavage assays for PNGM-1 mutants. usRNA1, usRNA9, and usDNA1 were incubated with 15 μM wild-type PNGM-1 or PNGM-1 mutant (H91A, H93A, H96A, H257A, or D95A) in the presence of (A) 10 mM MgCl_2_ or (B) MnCl_2_, at 50°C for 90 min, and the products were analyzed on a 20% polyacrylamide 8 M urea gel or a 20% polyacrylamide native gel. The native gel was used only for analysis of usDNA1 reactions with MnCl_2_. WT, wild type. Experiments in (A) and (B) were repeated at least three times and were reproducible.

To rule out the possibility that the observed RNase activity of PNGM-1 originates from unidentified contaminant RNases, we examined the five PNGM-1 mutants. These mutants contained single amino-acid substitutions of residues that were likely essential for RNase activity on usRNA1 and usRNA9. The PNGM-1 mutants (H91A, H93A, D95A, H96A, and H257A: five mutants with alanine replacements of His-48, His-50, Asp-52, His-53, and His-222 at structurally equivalent positions in Tm-tRNase Z, respectively) had a single substitution of alanine for histidine or aspartic acid. The corresponding amino-acid substitutions in Tm-tRNase Z are known to abolish its activity without Mn^2+^ ions [23]. All five PNGM-1 mutants showed little or no RNase activity in the presence of Mg^2+^ (Figure 5(A)). These results indicate that the observed RNase activity of PNGM-1 is genuine.

RNase activity was recovered, albeit inefficiently, in the presence of Mn^2+^ for mutants H91A, H93A, and H96A but not D95A and H257A (Figure 5(B)). This Mn^2+^-rescue phenomenon, which was first observed for the pre-tRNA cleavage reaction by Tm-tRNase Z [23], suggests that Asp-95 (Asp-52 in Tm-tRNase Z) and His-257 (His-222 in Tm-tRNase Z) are essential and directly contribute to proton transfer for the RNase activity of PNGM-1, further corroborating the RNase activity of PNGM-1. It has been suggested that the Mn^2+^-rescue is primarily due to the property that Mn^2+^ ions have higher affinity for either the active site or a nearby site, or both, than Mg^2+^ ions and that Mn^2+^ ions are able to be coordinated even in either the catalytic site or the nearby site, or both, even when they are distorted due to the lack of one of the important amino acids [23]. Interestingly, the turnover number (*k*_cat_) of H257A for all tested *β*-lactams decreased in comparison to that of wild-type PNGM-1 (Table 1), which suggests that H257 (as well as H91A, H93A, D95A, and H96A) of PNGM-1 plays an important role in both its RNase and *β*-lactamase activities.

### Cleavage site preference of the endoribonucleolytic activity of PNGM-1

**Figure 6.**
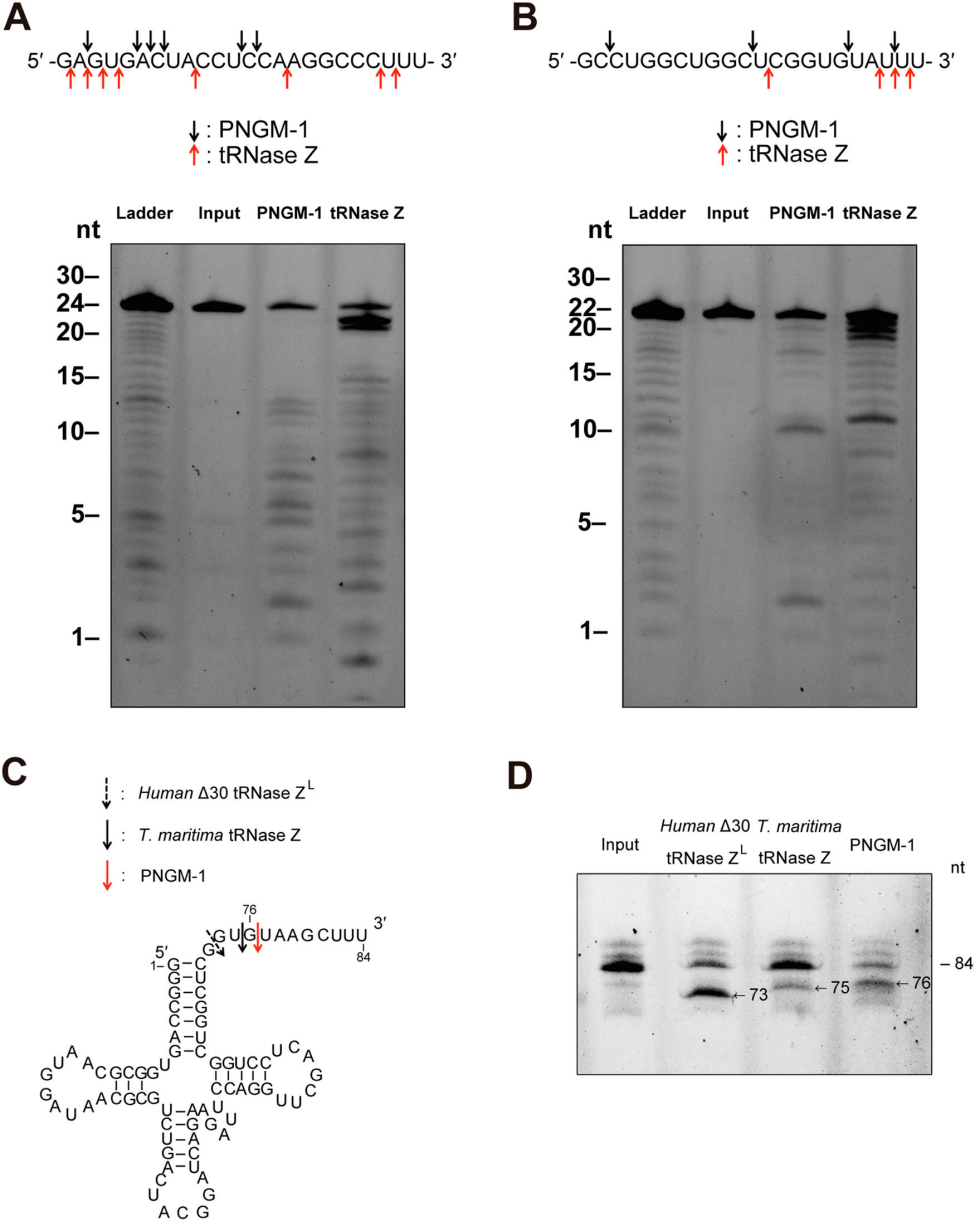
Cleavage site analysis for the RNA substrates usRNA1 and usRNA9 (A and B); and pre-tRNA processing assay (C and D). (A) usRNA1 and (B) usRNA9 were incubated at 50°C in the presence of 10 mM MgCl_2_ with 15 μM PNGM-1 for 90 min or in the presence of 10 mM MnCl_2_ with 1.5 μM Tm-tRNase Z for 30 min. Cleavage products were analyzed on a 20% polyacrylamide 8 M urea gel. Arrows indicate the major cleavage sites. (C) The secondary structure of human pre-tRNA^Arg^ and cleavage sites for human Δ30 tRNase Z^L^, Tm-tRNase Z, and PNGM-1. (D) The 84-nt 5′-fluorescein-labeled human pre-tRNA^Arg^ was incubated with 0.5 μM human Δ30 tRNase Z^L^ in the presence of 10 mM MgCl_2_ at 37°C for 30 min, with 1.5 μM Tm-tRNase Z at 37°C for 60 min or with 15 μM PNGM-1 at 50°C for 90 min. The products were analyzed on a 20% polyacrylamide 8 M urea gel. Experiments in (A)–(D) were repeated at least three times and were reproducible.

We examined the cleavage site preference of the endoribonucleolytic activity of PNGM-1 on usRNA1 and usRNA9. The cleavage reaction products for usRNA1 and usRNA9 were analyzed at nt resolutions, with corresponding substrate ladders, on a 20% polyacrylamide denaturing gel. The cleavage patterns of usRNA1 and usRNA9 by PNGM-1 were unique and different from those for Tm-tRNase Z (Figure 6(A) and (B)). PNGM-1 appears to have a tendency to cleave the RNA substrates between pyrimidine nucleotides.

### PNGM-1 can process a pre-tRNA substrate

To test whether PNGM-1 can process pre-tRNA similar to tRNase Z, the 84-nt human pre-tRNA^Arg^ was used as a pre-tRNA substrate (Figure 6(C)). The pre-tRNA^Arg^ was incubated with PNGM-1, Tm-tRNase Z or human Δ30 tRNase Z^L^, with cleavage products analyzed by denaturing polyacrylamide gel electrophoresis. PNGM-1 cleaved the pre-tRNA^Arg^ similar to Tm-tRNase Z and human Δ30 tRNase Z^L^ (Figure 6(D)). Major cleavage by PNGM-1 occurred after the 76th nt (3 nt downstream of the discriminator), whereas major cleavage by Tm-tRNase Z and human Δ30 tRNase Z^L^ occurred after the 75th nt and the discriminator (73rd nt), respectively, as expected [21,23]. Therefore, PNGM-1 is the first unique enzyme that has dual activity i.e. *β*-lactamase (MBL hydrolyzing all tested and clinically-used *β*-lactam antibiotics; Table 1) and tRNase Z activity.

## Discussion

MBLs hydrolyze almost all *β*-lactam antibiotics including carbapenems, thereby representing a critical antibiotic resistant threat [11,12]. The evolutionary origin of subclass B3 MBLs is currently unknown. Recently, we discovered a novel B3 MBL named PNGM-1, which was the first subclass B3 MBL enzyme obtained from a functional and bacterial metagenomic library of deep-sea sediments from the Edison seamount, which existed prior to the antibiotic era. Our phylogenetic analysis revealed that PNGM-1 was grouped with MBL fold proteins and was closest to the tRNase Zs of the MBL fold proteins with known enzymatic functions, which suggest that PNGM-1 gives us an insight into the evolutionary origin of subclass B3 MBLs. Although enzymatic functions of two MBL fold metallo-hydrolases (Pl and Ac) remain unknown, they showed higher sequence identity with PNGM-1 than tRNase Zs. These results suggest that Pl (or Ac) seems to have the characteristics of PNGM-1.

**Figure 7.**
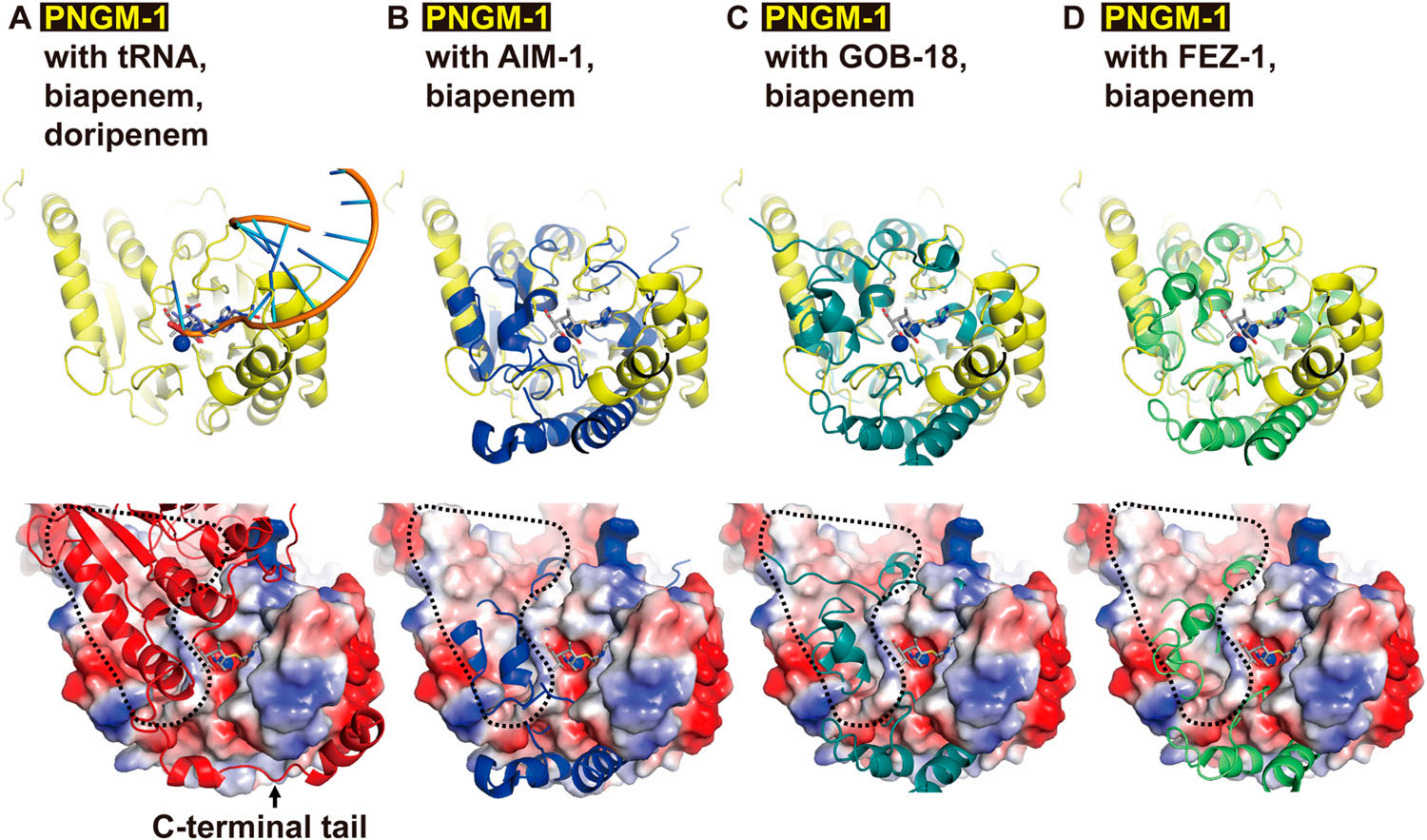
Structural comparison of PNGM-1 with subclass B3 MBLs. The structure of PNGM-1 (yellow) was superimposed with those of AIM-1 (blue, PDB entry 4AWZ), GOB-18 (pale green, PDB entry 5K0W), and FEZ-1 (lime, PDB entry 5W90) (top row). (A) PNGM-1 structure with superimposed tRNA, biapenem, and doripenem. (B) PNGM-1 structure with superimposed AIM-1 and biapenem. (C) PNGM-1 structure with superimposed GOB-18 and biapenem. (D) PNGM-1 structure with superimposed FEZ-1 and biapenem. The surface electrostatic potential of PNGM-1 (subunit A) is shown (bottom row). Structures of PNGM-1 (subunit B, red), AIM-1, GOB-18, and FEZ-1 are shown in cartoon representation. The main dimerization surface of PNGM-1 is marked with a black dashed line.

Our functional analyses of PNGM-1 showed that PNGM-1 has *β*-lactamase (MBL) and tRNase Z activity (Table 1 and Figures 3–7). We demonstrate the dual activity of PNGM-1, which strongly suggests that PNGM-1 has evolved from a tRNase Z [MBL fold protein with a single native activity and without *β*-lactamase activity (Table S7)]. This is in agreement with earlier reports suggesting that novel enzymes could evolve from an existing enzyme with single native activity through the recruitment of multiple functions (e.g. dual activity) [18,36]. It is also consistent with previous reports showing that (i) MBLs descended from a common ancestor (MBL fold protein) of the MBL superfamily [15] and (ii) the last common ancestor of the MBL superfamily is a phosphodiesterase, such as Ec-tRNase Z (PDB code: 2CBN), involved in tRNA processing [18,37].

All PNGM-1 molecules, eight monomers in the asymmetric unit, in the native structure have two zinc ions in the active site. Both zinc ions are tightly coordinated with the distance between 2.0 and 2.4 Å (Figure S2). When PNGM-1 structure is superimposed to Bs-tRNase Z, the position of the two zinc ions in both structures is conserved (Figure S3). We compared the PNGM-1 structure with carbapenem-bound subclass B3 MBL structures such as biapenem-bound CphA and doripenem-bound SMB-1 (Figure S4A and B). The superimposed biapenem and doripenem are almost in the same position as the 3’ end of tRNA close to the zinc ions in the active site (Figures 2 and 7). The substrate-binding pocket between PNGM-1 and subclass B3 MBLs is well conserved (Figure 7(B-D)). When we soaked doripenem in the crystals of PNGM-1, the doripenem with the hydrolyzed *β*-lactam ring was determined in the doripenem-bound PNGM-1 mutant (H257A) structure (Figure S5). However, the efficiency (*k*_cat_/*K_m_* for carbapenems [imipenem, meropenem, and ertapenem]) of PNGM-1 MBL activity is significantly lower than that of the single native MBL activity of AIM-1, GOB-18 or FEZ-1 by 3 or 4 orders (Tables 1 and S7) and lower than that of Tm-tRNase Z by 3 orders [21]. *β*-Lactam-containing antibiotics including carbapenems (substrates for MBLs) existed in natural environments [9] and natural antibiotics appear to exist in the biosphere billions years ago [38,39]. It means that the B3 MBL activity of PNGM-1 is a promiscuous activity and subclass B3 MBLs is thought to have evolved from PNGM-1. It is consistent with the Jensen conceptualized enzyme evolution showing that newly specialized enzymes are generated from promiscuous activities [18,40].

Based on our results, we suggest that subclass B3 MBLs arose through an evolutionary trajectory (tRNase Z → PNGM-1→ subclass B3 MBLs) and the origin of subclass B3 MBLs is a tRNase Z. The evolutionary trajectory would be strongly supported if two MBL fold metallo-hydrolases (Pl and Ac) might have the dual activity of PNGM-1. If a B1/B2 MBL with dual activity, like PNGM-1, could be identified in the future, we would be able to understand the evolutionary origin of subclass B1/B2 MBLs, as MBLs have evolved independently twice (B1/B2 and B3 subclasses) billions years ago [15,18,41]. These evolutionary processes help us to understand where MBL genes came from and predict the future evolution of MBL genes, as previously described [39]. In addition, the molecular evolutionary process (dimer interfacial deformation to prevent dimerization of an ancestral protein such as a tRNase Z, dimer) provides a new strategy to develop novel enzymes (e.g. class B MBLs, monomer). To date novel enzymes have rationally been designed by two main approaches: (i) grafting of a reactive catalytic motif into a protein scaffold; and (ii) amino acid substitutions to improve catalytic activity or to create new catalytic activities [42].

## Supplementary Material

Supplemental Material

## Data Availability

Coordinates for the PNGM-1 atomic model have been deposited in the Protein Data Bank under the accession codes 6J4N (native) and 6JKW (SeMet).
