# Supplementary material for: Dual activity of PNGM-1 pinpoints the evolutionary origin of subclass B3 metallo-β-lactamases: a molecular and evolutionary study
Source: Emerg Microbes Infect. 2019 Nov 21;8(1):1688–700. doi: 10.1080/22221751.2019.1692638 (PMC6882493; doi:10.1080/22221751.2019.1692638)
Supplement: Supplemental Material [file TEMI_A_1692638_SM7918.zip › Supplemental_data_TEMI_2019_0551.R1_final.docx]

**Supplementary Information**

**Supplementary Figures**


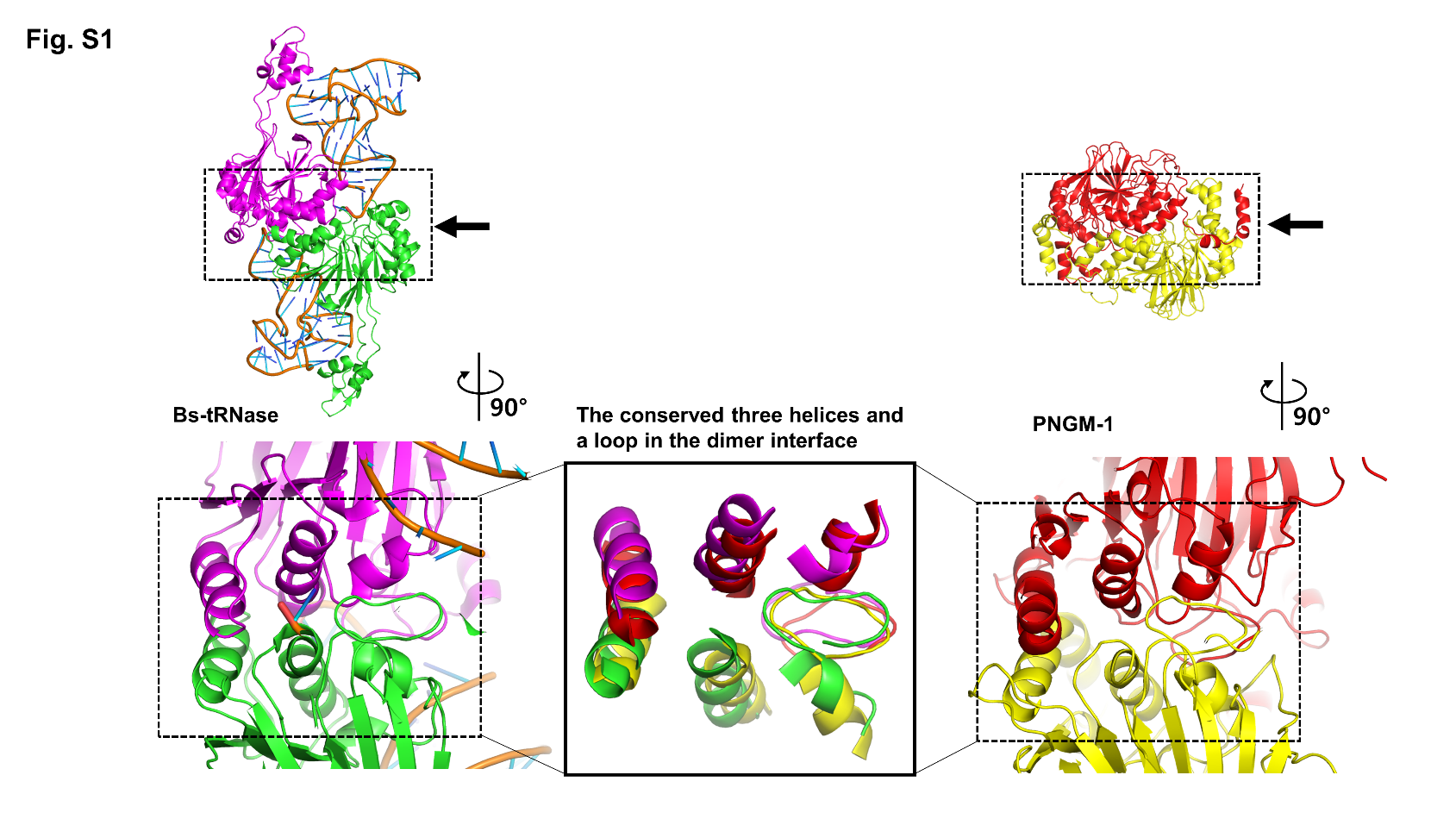


**Figure S1.** The conserved dimer interface of Bs-tRNase Z and PNGM-1. The upper figures show the same orientation with Figure 2 and the lower figures show the side view (arrow) of the same structures. The dimer interface consists of three helices and a loop: three helices of residues 43-49, 70-82, and 95-106 and a loop of residues 9-18 in Bs-tRNase Z and three helices of residues 71-77, 98-110, and 132-143 and a loop of residues 39-48 in PNGM-1.


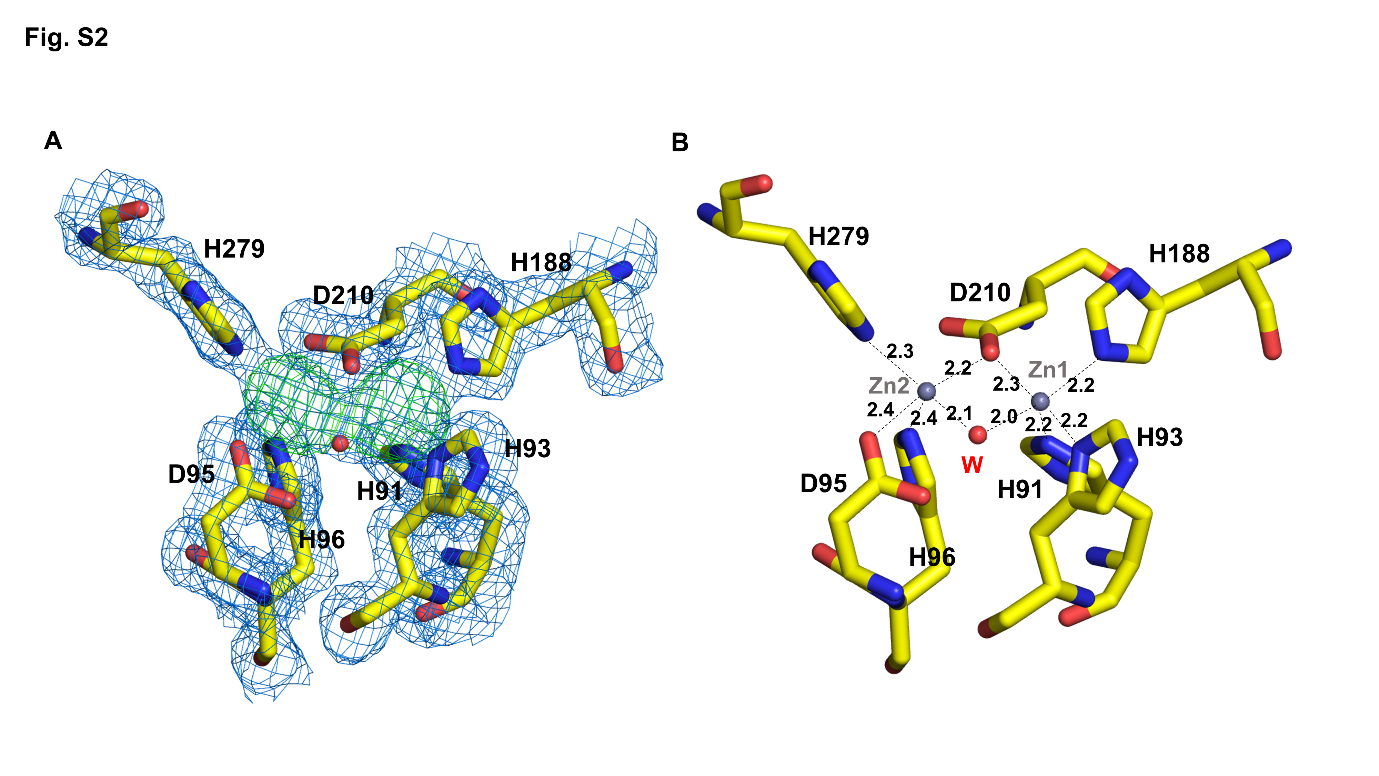


**Figure S2.** The active site structure of PNGM-1. (A) The 2FoFc (blue; contoured at the 1σ level) and FoFc (green; contoured at the 5σ level) maps of the active site of PNGM-1 without two zinc ions. (B) The coordination of the two bound zinc ions in PNGM-1.


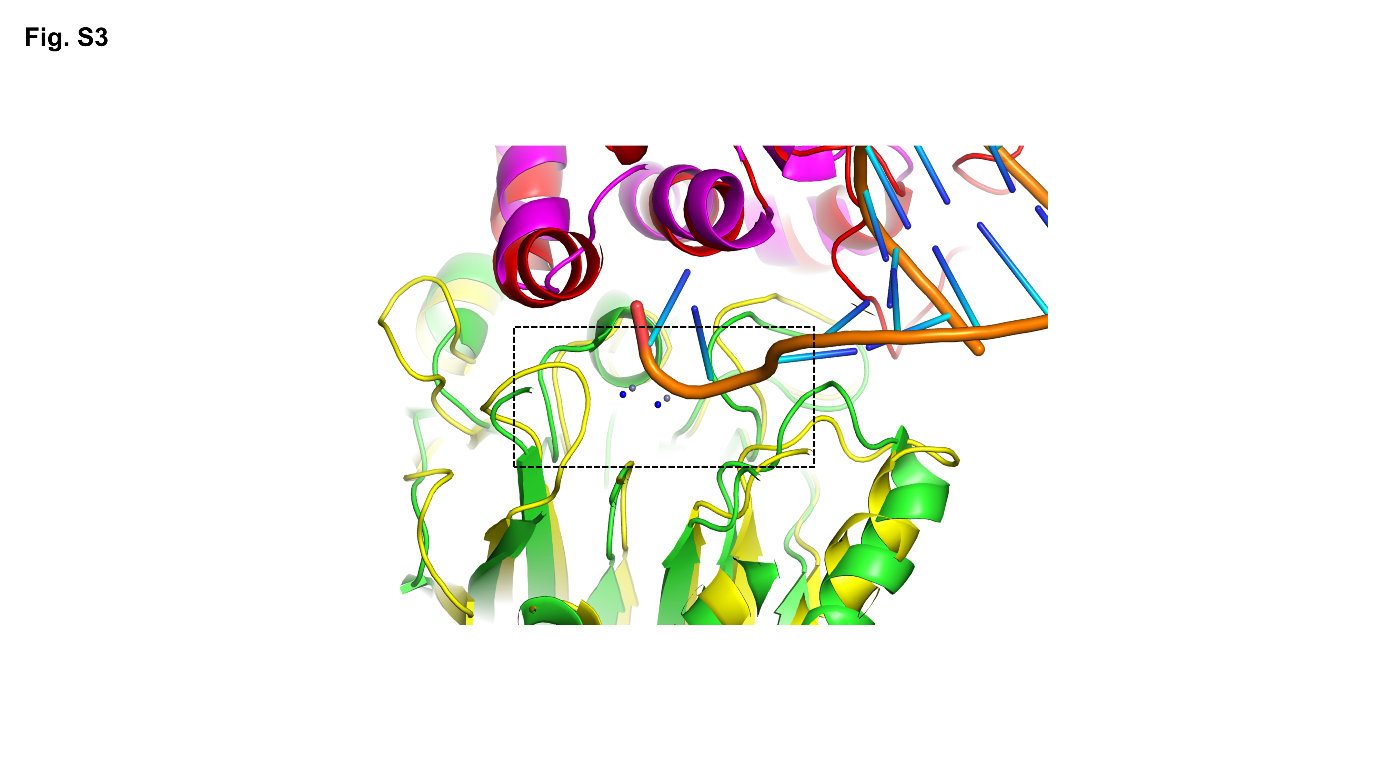


**Figure S3.** The superimposed structures of Bs-tRNase Z and PNGM-1 active site. The colors are same as in Figure 2. The two zinc ions of Bs-tRNase Z are shown in blue and those of PNGM-1 in grey.


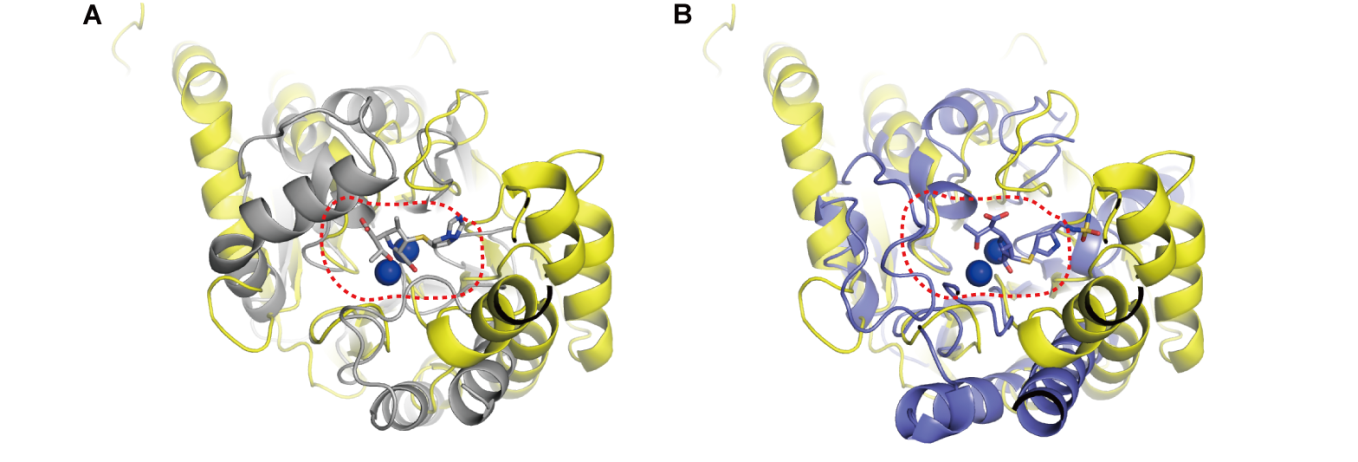


**Figure S4.** Structure comparison of PNGM-1 with a carbapenem-bound MBL. PNGM-1 (yellow) is superimposed with (A) biapenem-bound CphA (grey, PDB entry 1X8I) and (B) doripenem-bound SMB-1 (pale purple, PDB entry 5B15). The two zinc ions in the active site are shown (blue sphere). The substrate-binding pocket is marked with a red dashed line.


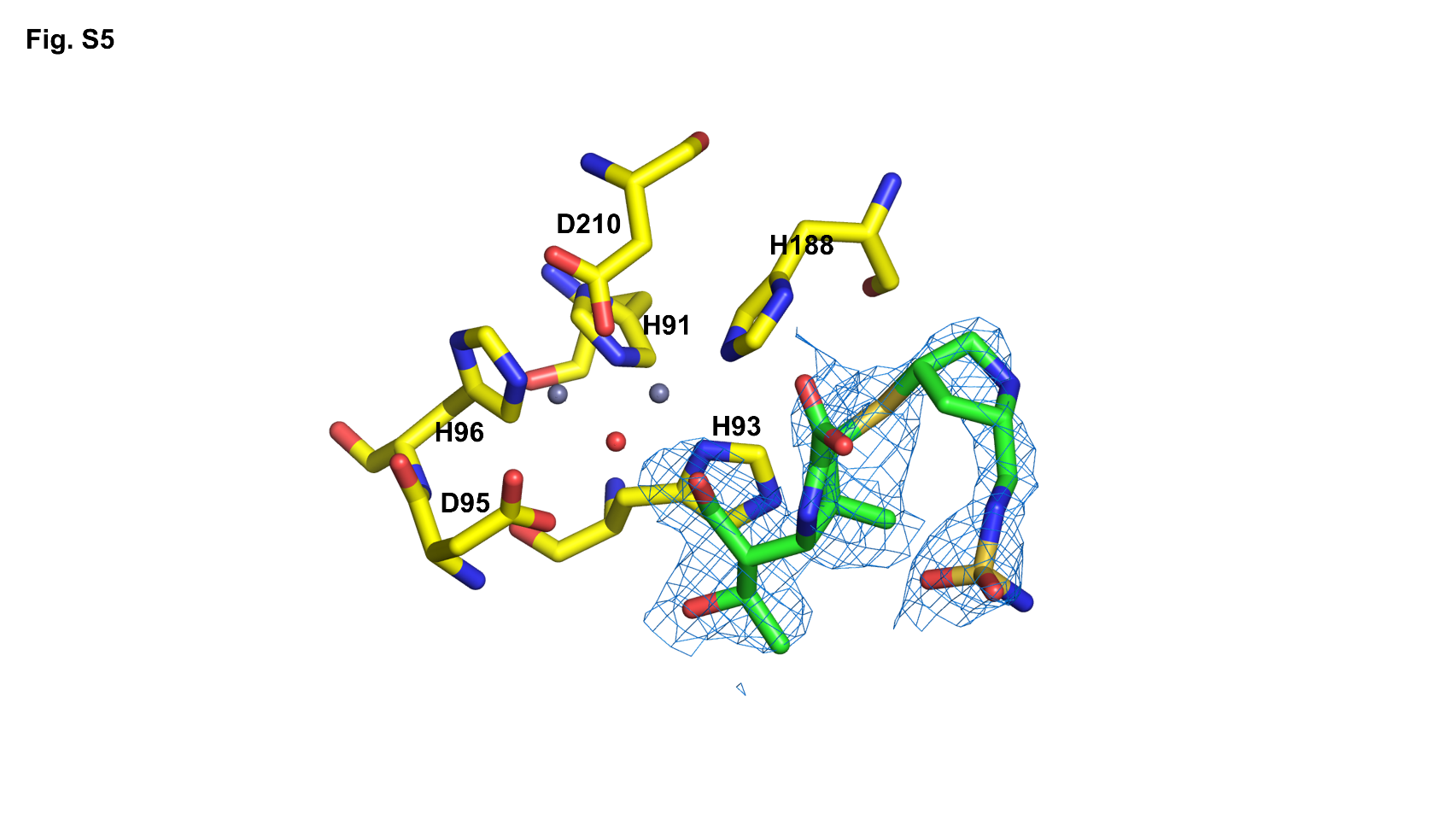


**Figure S5.** The 2FoFc map of doripenem (green; the 3-letter code of DRW) bound in PNGM-1 mutant (H257A) and the active site structure of doripenem-bound H257A. The 2FoFc (blue; contoured at the 1σ level) map is shown in blue. The hydrolyzed form of the β-lactam ring of doripenem is shown.

**Supplementary Tables**

**Table S1.** Strains and plasmids used in this study.

| Strains and plasmids | Phenotype, genotype and/or characteristics | Source  (or reference) |
| --- | --- | --- |
| Strains |  |  |
| *E. coli* BL21(DE3) | F^-^*ompT hsdS_B_*(r_B_^-^m_B_^-^) *gal dcm* (DE3) | Invitrogen |
| Plasmids |  |  |
| pET-28a(+) | Expression vector, kanamycin^r^ | Novagen |
| pET-30a(+) | Expression vector, kanamycin^r^ | Novagen |
| pET-28a(+)/His_6_-PNGM-1 | The *bla*_PNGM-1_ gene was cloned into the pET-28a(+) vector, encoding N-terminal His_6_-tag | Park *et al.* (2018)[13] |
| pET-28a(+)/His_6_-PNGM-1 (H91A) | H91 in PNGM-1 was replaced by alanine by site-directed mutagenesis | This study |
| pET-28a(+)/His_6_-PNGM-1 (H93A) | H93 in PNGM-1 was replaced by alanine by site-directed mutagenesis | This study |
| pET-28a(+)/His_6_-PNGM-1 (D95A) | D95 in PNGM-1 was replaced by alanine by site-directed mutagenesis | This study |
| pET-28a(+)/His_6_-PNGM-1 (H96A) | H96 in PNGM-1 was replaced by alanine by site-directed mutagenesis | This study |
| pET-28a(+)/His_6_-PNGM-1 (H257A) | H257 in PNGM-1 was replaced by alanine by site-directed mutagenesis | This study |
| pET-30a(+)/His_6_-AIM-1 | The *bla*_AIM-1_ gene was cloned into the pET-30a(+) vector | This study |
| pET-30a(+)/His_6_-GOB-18 | The *bla*_GOB-18_ gene was cloned into the pET-30a(+) vector | This study |
| pET-30a(+)/His_6_-FEZ-1 | The *bla*_FEZ-1_ gene was cloned into the pET-30a(+) vector | This study |
| pET-30a(+)/His_6_-Bs-tRNase Z | The tRNase Z gene from *Bacillus subtilis* was cloned into the pET-30a(+) vector | This study |
| pET-30a(+)/His_6_-Ec-tRNase Z | The tRNase Z gene from *Escherichia coli* was cloned into the pET-30a(+) vector | This study |
| pET-30a(+)/His_6_-Tm-tRNase Z | The tRNase Z gene from *Thermotoga maritima* was cloned into the pET-30a(+) vector | This study |

r: resistant.

**Table S2.** Primers used in this study.

| Name | Sequence (5′→3′) |
| --- | --- |
| Primers for site-directed mutagenesis |  |
| PNGM-1-H91A-F*^a^* | 5'–AAGATTTTTCTGACGGCCTTGCACACCGACCAC–3' |
| PNGM-1-H91A-R*^b^* | 5'–GTGGTCGGTGTGCAAGGCCGTCAGAAAAATCTT–3' |
| PNGM-1-H93A-F | 5'–TTTCTGACGCATTTGGCCACCGACCACTGGGGC–3' |
| PNGM-1-H93A-R | 5'–GCCCCAGTGGTCGGTGGCCAAATGCGTCAGAAA–3' |
| PNGM-1-D95A-F | 5'–ACGCATTTGCACACCGCCCACTGGGGCGACCTG–3' |
| PNGM-1-D95A-R | 5'–CAGGTCGCCCCAGTGGGCGGTGTGCAAATGCGT–3' |
| PNGM-1-H96A-F | 5'–CATTTGCACACCGACGCCTGGGGCGACCTGGTG–3' |
| PNGM-1-H96A-R | 5'–CACCAGGTCGCCCCAGGCGTCGGTGTGCAAATG–3' |
| PNGM-1-H257A-F | 5'–ATCAATCTGGACTTTGCCACCTCAGCGCAATCC–3' |
| PNGM-1-H257A-R | 5'–GGATTGCGCTGAGGTGGCAAAGTCCAGATTGAT–3' |
| Primers for cloning |  |
| *Nde*I-His-EK-AIM-1-F | 5'–ATA**CATATGCATCATCATCATCATCAT***GACGACGACGACAAG* TCGGATGCTCCAGCCTCAAGAGGA–3' |
| *Xho*I-AIM-1-R | 5'–GAG**CTCGAG**TCAAGGCCGAGCACCACTAGAC–3' |
| *Nde*I-His-EK-GOB-18-F | 5'–ATA**CATATGCATCATCATCATCATCAT***GACGACGACGACAAG* GCTCAGGTAGTAAAAGAACCTGAAAATAT–3' |
| *Xho*I- GOB-18-R | 5'–CAG**CTCGAG**TTATTTCTTTATTGCATTCAGCACAG–3' |
| *Nde*I-His-EK-FEZ-1-F | 5'–ATA**CATATGCATCATCATCATCATCAT***GACGACGACGACAAG* GCTTATCCAATGCCTAACCCC–3' |
| *Xho*I-FEZ-1-R | 5'–CAG**CTCGAG**TTATTTATCTTGGGAATCTTTTTTTATTTTGTTGA  GAT–3' |
| *Nde*I-His-EK-Bs-tRNaseZ-F | 5'–ATA**CATATGCATCATCATCATCATCAT***GACGACGACGACAAG* GAGTTATTATTCTTGGGTACTGGTGCGG–3' |
| *Xho*I-Bs-tRNaseZ-R | 5'–GAG**CTCGAG**TCAACCGCGGGGAACGTTGACT–3' |
| *Nde*I-His-EK-Ec-tRNaseZ-F | 5'–ATA**CATATGCATCATCATCATCATCAT***GACGACGACGACAAG* GAATTAATTTTTTTAGGTACTTCAGCCGG–3' |
| *Xho*I-Ec-tRNaseZ-R | 5'–CAG**CTCGAG**TTAAACGTTAAACACGGTGAAATCATTCGCC–3' |
| *Nde*I-His-EK-Tm-tRNaseZ-F | 5'–ATA**CATATGCATCATCATCATCATCAT***GACGACGACGACAAG* AACATAATCGGCTTCAGCAAAG –3' |
| *Xho*I-Tm-tRNaseZ-R | 5'–GAG**CTCGAG**TCACATTTCAAATACTTTTCTCGGG–3' |

The positions of the mutated codons are underlined. Restriction sites appear in bold. The underlined and bolded bases indicate the hexahistidine tag, and italic bases indicate the enterokinase recognition site.

*^a^*F, forward.

*^b^*R, reverse.

**Table S3.** List of the representative types of metallo-β-lactamases (MBLs) and structurally representative MBL fold proteins used to construct the phylogenetic trees for PNGM-1.

| Subclass B1  MBLs | | Subclasses B2 and B3 MBLs | | MBL fold proteins | | |
| --- | --- | --- | --- | --- | --- | --- |
| Name | Accession no.*^a^* | Name | Accession no.*^a^* | Name | Accession no.*^a^* or UniProt ID*^b^* | Description |
| ANA-1 | WP_041449074 | CphA-1 | CAA40386 | Bs-tRNase Z | P54548*^b^* | tRNase Z or Ribonuclease Z |
| BcII-1 | AAA22276 | SFH-1 | WP_024531368 | Tm-tRNase Z | AKE26778*^a^* |  |
| BlaB-1 | AAF89154 | ImiS | CAA71441 | Ec-tRNase Z | P0A8V0^b^ |  |
| CfiA | AAA22907 | ImiH | CAD69003 | Tm | NP_228022*^a^* | Zn-dependent hydrolase |
| CGB-1 | AAL55263 | PNGM-1 | AWN09461 | Tm-1 | Q9X207*^b^* | Zn-dependent hydrolase |
| DIM-1 | AGC92784 | AIM-1 | CAQ53840 | Tm-Lac | Q9WZZ6*^b^* | Lactonase |
| EBR-1 | AAN32638 | ALG6-1 | APR64488 | AiiA | P0CJ63*^b^* | N-Acyl Homoserine Lactone Hydrolase |
| ECV-1 | AGA78874 | ALG11-1 | APR64489 | PDLA | Q988B9*^b^* | 4-pyridoxolactonase |
| ElBla2-1 | ABC63608 | BJP-1 | BAC51495 | SdsA1 | AAG04129*^a^* | Alkylsulfatase |
| FIA-1 | WP_041258349 | CAR-1 | AIA71664 | ATSD | Q9C8L4*^b^* | Sulfur dioxygenase |
| FIM-1 | AFV91534 | CAU-1 | CAC87665 | CbpE | CAC29434*^a^* | Teichoic acid phosphorylcholine esterase |
| GIM-1 | ALO69078 | CPS-1 | AJP77054 | Pah | AAP06948*^a^* | Methyl parathion hydrolase |
| GRD23-1 | APR64493 | CRD3-1 | APR64487 | Gox | Q16775*^b^* | Glyoxalase II |
| HMB-1 | AMY61250 | DHT2-1 | APR64485 | MTH1203 | WP_010876827*^a^* | β-CASP metallo-β-lactamase family nuclease |
| IMP-1 | ABK27309 | EAM-1 | AFN85388 | Pl | WP_146397269*^a^* | MBL fold metallo-hydrolase |
| IND-1 | AAD20273 | ECM-1 | AFN85387 | Ac | TDI33721*^a^* | MBL fold metallo-hydrolase |
| JOHN-1 | AAK38324 | EFM-1 | AFN85384 |  |  |  |
| KHM-1 | BAF91108 | ELM-1 | AFN85386 |  |  |  |
| MOC-1 | ANJ59787 | ESP-1 | AJP77085 |  |  |  |
| MUS-1 | AAN63647 | EVM-1 | AFN85385 |  |  |  |
| MYO-1 | WP_081048762 | FEZ-1 | CAB96921 |  |  |  |
| MYX-1 | ABF86854 | GOB-1 | AAF04458 |  |  |  |
| ` | AHM26723 | L1-1 | CAA52968 |  |  |  |
| ORR-1 | WP_109545042 | LRA3-1 | ACH58987 |  |  |  |
| PEDO-3 | AJP77076 | LRA7-1 | ACH58998 |  |  |  |
| PST-1 | WP_043942497 | LRA8-1 | ACH58988 |  |  |  |
| SFB-1 | AAT90847 | LRA12-1 | ACH58990 |  |  |  |
| SHD-1 | ABE54111 | LRA17-1 | ACH58994 |  |  |  |
| SHN-1 | ABE56430 | LRA19-1 | ACH59005 |  |  |  |
| SIM-1 | AER61546 | LRA2-1 | ACH58985 |  |  |  |
| SLB-1 | AAT90846 | MEMA1-1 | KY705336 |  |  |  |
| SPM-1 | AAR15341 | MIM-1 | AIT78529 |  |  |  |
| SPN79-1 | APR64486 | MSI-1 | AJP77057 |  |  |  |
| SPS-1 | ADK81930 | PEDO-1 | AJP77059 |  |  |  |
| STA-1 | WP_109545039 | PLN-1 | KIO75746 |  |  |  |
| TTU-1 | ACR12883 | POM-1 | ABY56045 |  |  |  |
| TMB-1 | CBY88906 | RM3 | AGU01679 |  |  |  |
| TUS-1 | EKB08120 | SAG-1 | AFV00127 |  |  |  |
| VIM-1 | CAC35170 | SMB-1 | BAL14456 |  |  |  |
| ZOG-1 | CAZ94871 | SPG-1 | AJP77080 |  |  |  |
|  |  | SPR-1 | ABV42357 |  |  |  |
|  |  | THIN-B | CAC33832 |  |  |  |

*^a^*NCBI GenBank database accession number (http://www.ncbi.nlm.nih.gov/).

*^b^*UniProt is a freely accessible database of protein sequence and functional information (https://www.uniprot.org/).

**Table S4.** Data collection and refinement statistics.

| **Data collection** | SeMet | Native | Doripenem-bound  H257A |
| --- | --- | --- | --- |
| Space group | *P*2_1_ | *P*2_1_ | *P*2_1_ |
| Unit-cell parameters |  |  |  |
| a, b, c (Å) | 121.9, 83.1, 162.8 | 122.3, 83.0, 163.5 | 79.6, 143.3, 79.5 |
| α, β, γ (°) | 90, 110.2, 90 | 90, 110.6, 90 | 90, 111.9, 90 |
| Resolution (Å) | 50.0 – 2.3 (2.34 – 2.30) | 50.0 – 2.1 (2.14 – 2.10) | 50.0 – 2.58 (2.62 – 2.58) |
| Total reflections | 1,018,484 | 1,318,149 | 190,444 |
| Unique reflections | 135,186 | 175,405 | 49,907 |
| Completeness (%) | 98.1 (86.8) | 98.2 (97.5) | 95.2 (87.6) |
| Multiplicity | 7.5 (6.9) | 7.5 (7.4) | 2.1 (1.9) |
| 〈*I/σ*(*I*)〉 | 46.8 (11.3) | 47.7 (15.5) | 7.5 (3.0) |
| *R*_merge_ (%) | 12.2 (34.2) | 9.0 (22.7) | 23.6 (43.8) |
|  |  |  |  |
| **Refinement** |  |  |  |
| Resolution (Å) | 33.3-2.3 (2.37-2.29) | 34.3–2.1 (2.17–2.10) | 40.1–2.57 (2.67–2.56) |
| Used reflections | 134,467 (12,544) | 175,021 (17,252) | 175,021 (17,252) |
| Macromolecules /  asymmetric unit | 8 | 8 | 4 |
| *R*_work_ / *R*_free_ (%) | 21.3/26.8 | 19.2/24.3 | 25.7/28.7 |
| No. of atoms | 23,910 | 24,786 | 10,626 |
| Protein | 23,464 | 23,464 | 10,395 |
| Water | 430 | 1,306 | 169 |
| Zn^2+^ ion | 16 | 16 | 8 |
| Average B-factor (Å) | 32.9 | 25.5 | 23.1 |
| Protein | 33.0 | 25.5 | 23.1 |
| Water | 26.2 | 25.2 | 11.9 |
| Zn^2+^ ion | 31.4 | 24.1 | 19.8 |
| Doripenem |  |  | 71.5 |
| Protein geometry (%) |  |  |  |
| Poor rotamers | 5.71 | 4.35 | 7.27 |
| Favored rotamers | 87.50 | 89.28 | 81.96 |
| Ramachandran outliers | 1.05 | 0.95 | 2.87 |
| Ramachandran favored | 93.95 | 95.00 | 87.68 |
| Cβ deviations >0.25Å | 0.37 | 0.22 | 0.00 |
| Bad bonds | 0.00 | 0.01 | 0.01 |
| Bad angles | 0.03 | 0.07 | 0.23 |

Dataset was collected from a single crystal. Data collection data was previously published [29]. Values in parentheses are for the shell with the highest-resolution. *R*_merge =_ $\sum_{hkl} \sum_{i} \left| \left( I_{i}\left( hkl \right) \right)-\left\langle I\left( hkl \right) \right\rangle\right|/\sum_{hkl} \sum_{i} I_{i}\left( hkl \right),$ where $I_{i}\left( hkl \right)$ is the mean intensity of the *i*th observation of symmetry-related reflections *hkl*. *R*_work_ = $\sum_{hkl} \left| \left| F_{obs} \right|-\left| F_{calc} \right| \right|/\sum_{hkl} \left| F_{obs} \right|,$ where $F_{calc}$ is the calculated protein structure factor from the atomic model (*R*_free_ was calculated as *R*_work_ with a randomly selected 5% of the reflections). Protein geometry was analyzed by MolProbity (molprobity.biochem.duke.edu). Several residues, located at the end of β-strand or in a loop, showed non-favorable dihedral angles.

**Table S5.** The effect of NaCl on β-lactam hydrolysis activity (V_max_) of PNGM-1.

| NaCl (mM) | Activity (µM·s^-1^) | | |  |
| --- | --- | --- | --- | --- |
|  | Benzylpenicillin |  | Cephalothin |  |
| 0 | 0.132 |  | 0.229 |  |
| 50 | 0.145 |  | 0.252 |  |
| 100 | 0.145 |  | 0.252 |  |
| 250 | 0.145 |  | 0.252 |  |
| 500 | 0.145 |  | 0.252 |  |
| 1,000 | 0.145 |  | 0.252 |  |
| 2,000 | 0.145 |  | 0.252 |  |

**Table S6.** Comparison of PNGM-1 structure against all structures in the Protein Data Bank (PDB).

| Organism | Chain | Z*^a^* | rmsd*^b^* | lali*^c^* | nres*^d^* | %id*^e^* | Protein |
| --- | --- | --- | --- | --- | --- | --- | --- |
| *Bacillus subtilis* | 4GCW-A | 29.7 | 2.2 | 251 | 307 | 20 | Bs-tRNase Z |
| *Escherichia coli* | 2CBN-A | 29.4 | 2 | 249 | 306 | 21 | Ec-tRNase Z |
| *Thermotoga*  *maritima* | 2E7Y-A | 22.2 | 2.5 | 220 | 272 | 16 | Tm-tRNase Z |
| *Pseudomonas aeruginosa* | 4AWZ-A | 10.0 | 3.7 | 166 | 269 | 13 | AIM-1 |
| *Elizabethkingia meningosep* | 5K0W-A | 9.8 | 3.3 | 163 | 270 | 13 | GOB-18 |
| *Legionella*  *gormanii* | 5W90-A | 9.6 | 3.2 | 161 | 263 | 9 | FEZ-1 |

A total of 1,389 hits (≥ Dali Z-score 2 among 154,939 structures in PDB, as of 19 August 2019) were found from the DALI search. Bs-tRNase Z had the highest Z-score (29.7) and succinyl-CoA synthetase had the lowest Dali Z-score (2.0). Similarities with Dali Z-scores lower than 2 are spurious.

*^a^*Dali Z-score, the statistical significance of the similarity between the protein-of-interest and other neighboring proteins.

*^b^*Root Mean Square Distance (rmsd), root-mean-square deviation of C-α atoms in the least-squares superimposition of the structurally equivalent C-α atoms.

*^c^*lali, the number of structurally equivalent residues.

*^d^*nres, the total number of amino acids in the hit protein.

*^e^*%id, the percentage of identical amino acids over structurally equivalent residues.

Representative comparison results are shown in this Table and all results are presented in Dataset 1.

**Table S7.** Kinetic parameters of AIM-1, GOB-18, FEZ-1, and three tRNase Zs for various β-lactams.

| Substrate and parameter | | AIM-1 | GOB-18 | FEZ-1 | Ec-tRNase Z | Bs-tRNase Z | Tm-tRNase Z |
| --- | --- | --- | --- | --- | --- | --- | --- |
| Cefoxitin | |  |  |  |  |  |  |
|  | *K*_m_ (μM) | 10.1 ± 0.1 | 12.5 ± 0.1 | 9.2 ± 0.1 | ND*^a^* | ND | ND |
|  | *k*_cat_ (s^–1^) | 4.725 ± 0.001 | 2.628 ± 0.001 | 1.564 ± 0.001 | ND | ND | ND |
|  | *k*_cat_/*K*_m_ (M^–1^ s^–1^) | (4.7 ± 0.2) × 10^5^ | (2.1 ± 0.2) × 10^5^ | (1.7 ± 0.1) × 10^5^ | ND | ND | ND |
| Ceftazidime | |  |  |  |  |  |  |
|  | *K*_m_ (μM) | 8.3 ± 0.2 | 10.3 ± 0.2 | 7.6 ± 0.2 | ND | ND | ND |
|  | *k*_cat_ (s^–1^) | 3.718 ± 0.001 | 2.067 ± 0.001 | 3.511 ± 0.001 | ND | ND | ND |
|  | *k*_cat_/*K*_m_ (M^–1^ s^–1^) | (4.5 ± 0.2) × 10^5^ | (2.0 ± 0.2) × 10^5^ | (4.6 ± 0.2) × 10^5^ | ND | ND | ND |
| Cefotaxime | |  |  |  |  |  |  |
|  | *K*_m_ (μM) | 5.4 ± 0.1 | 6.7 ± 0.1 | 4.9 ± 0.1 | ND | ND | ND |
|  | *k*_cat_ (s^–1^) | 2.157 ± 0.002 | 1.2 ± 0.003 | 1.205 ± 0.002 | ND | ND | ND |
|  | *k*_cat_/*K*_m_ (M^–1^ s^–1^) | (4.0 ± 0.1) × 10^5^ | (1.8 ± 0.1) × 10^5^ | (2.5 ± 0.1) × 10^5^ | ND | ND | ND |
| Meropenem | |  |  |  |  |  |  |
|  | *K*_m_ (μM) | 4.6 ± 0.1 | 5.7 ± 0.1 | 4.2 ± 0.1 | ND | ND | ND |
|  | *k*_cat_ (s^–1^) | 15.409 ± 0.0001 | 8.568 ± 0.001 | 0.773 ± 0.001 | ND | ND | ND |
|  | *k*_cat_/*K*_m_ (M^–1^ s^–1^) | (3.3 ± 0.1) × 10^6^ | (1.5 ± 0.1) × 10^6^ | (1.8 ± 0.1) × 10^5^ | ND | ND | ND |
| Imipenem | |  |  |  |  |  |  |
|  | *K*_m_ (μM) | 3.8 ± 0.1 | 4.8 ± 0.1 | 3.5 ± 0.1 | ND | ND | ND |
|  | *k*_cat_ (s^–1^) | 11.128 ± 0.0001 | 6.188 ± 0.003 | 0.763 ± 0.001 | ND | ND | ND |
|  | *k*_cat_/*K*_m_ (M^–1^ s^–1^) | (2.9 ± 0.2) × 10^6^ | (1.3 ± 0.2) × 10^6^ | (2.2 ± 0.1) × 10^5^ | ND | ND | ND |
| Ertapenem | |  |  |  |  |  |  |
|  | *K*_m_ (μM) | 5.0 ± 0.1 | 6.3 ± 0.1 | 4.6 ± 0.1 | ND | ND | ND |
|  | *k*_cat_ (s^–1^) | 12.376 ± 0.0001 | 6.882 ± 0.002 | 0.667 ± 0.001 | ND | ND | ND |
|  | *k*_cat_/*K*_m_ (M^–1^ s^–1^) | (2.5 ± 0.2) × 10^6^ | (1.1 ± 0.2) × 10^6^ | (1.5 ± 0.2) × 10^5^ | ND | ND | ND |

*^a^*ND, not detectable.

Data are mean ± s.d. of three assays.
